# Litoralimycins A and B, New Cytotoxic Thiopeptides from *Streptomonospora* sp. M2

**DOI:** 10.3390/md18060280

**Published:** 2020-05-26

**Authors:** Shadi Khodamoradi, Marc Stadler, Joachim Wink, Frank Surup

**Affiliations:** 1 Microbial Strain Collection, Helmholtz-Centre for Infection Research (HZI), Inhoffenstr. 7, 38124 Braunschweig, Germany; shadi.khodamoradi@helmholtz-hzi.de; 2 German Centre for Infection Research (DZIF), partner site Hannover-Braunschweig, 38124 Braunschweig, Germany; marc.stadler@helmholtz-hzi.de; 3 Microbial Drugs Department, Helmholtz-Centre for Infection Research (HZI), Inhoffenstr. 7, 38124 Braunschweig, Germany

**Keywords:** thiopeptide antibiotic, screening, structure elucidation, natural products, rare actinobacteria

## Abstract

*Streptomonospora* sp. M2 has been isolated from a soil sample collected at the Wadden Sea beach in our ongoing program aimed at the isolation of rare Actinobacteria, ultimately targeting the discovery of new antibiotics. Because crude extracts derived from cultures of this strain showed inhibitory activity against the indicator organism *Bacillus subtilis*, it was selected for further analysis. HPLC–MS analysis of its culture broth revealed the presence of lipophilic metabolites. The two major metabolites of those were isolated by preparative reversed-phase HPLC and preparative TLC. Their planar structures were elucidated using high-resolution electrospray ionization mass spectrometry (HRESIMS), 1D and 2D NMR data as new thiopeptide antibiotics and named litoralimycin A (**1**) and B (**2**). Although rotating frame nuclear Overhauser effect spectroscopy (ROESY) data established a Z configuration of the Δ^21,26^ double bond, the stereochemistry of C-5 and C-15 were assigned as S by Marfey’s method after ozonolysis. The biological activity spectrum of **1** and **2** is highly uncommon for thiopeptide antibiotics, since they showed only insignificant antibacterial activity, but **1** showed strong cytotoxic effects.

## 1. Introduction

New antibiotics in general and new types of antibiotics in particular are urgently needed to counter the increasing number of pathogenic bacteria resistant against present antibiotics [1]. Traditionally, actinobacteria have been the most prolific sources of novel antibiotics scaffolds, because many of the most important antimicrobials, such as β-lactames, tetracyclines, rifamycins, aminoglycosides, macrolides and glycopeptides, were discovered from them [2]. However, high rates of rediscovery of known compounds are observed when screening traditional producers, and the discovery of new molecules is getting more and more challenging. Therefore, current screening programs concentrate on discovering and isolating rare genera of microorganisms. Rare actinobacteria are regarded as actinomycete strains whose isolation frequency is much lower than that of *Streptomyces* spp. isolated by conventional methods. These rare actinobacteria are assessed as a potential storehouse for novel antibiotics due to their unique potential to produce novel metabolites [3,4].

**Figure 1:**
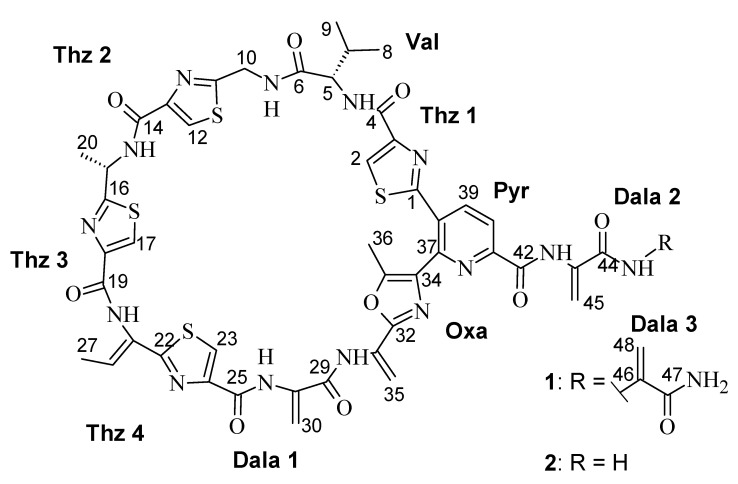
Chemical structure of **1** and **2**.

The approach of utilizing rare organisms is accompanied by the screening of organisms from underexplored environments. Rare actinomycetes are widely distributed in terrestrial and aquatic ecosystems and the number of isolated genera and species is quickly increasing due to recently developed taxonomically selective isolation procedures, cultivation methods and genetic techniques [5]. We isolated the new strain *Streptomonospora* sp. M2 from a Wadden Sea sample collected at a beach near Cuxhaven, Germany, which is an underexplored environment. Since crude extracts of *Streptomonospora* sp. M2 showed inhibitory activity against Gram-positive indicator organisms including *Micrococcus luteus, Staphylococcus aureus* and *Bacillus subtilis*, the strain was selected for a detailed analysis of its bioactive secondary metabolites, yielding the isolation and structure elucidation of two new thiopeptide antibiotics (Figure 1) with an uncommon activity profile.

## 2. Results

### 2.1. Screening

By a fractionation of the crude extracts in 96-well plates, it was possible to link the antibacterial activity to a region containing two major peaks (Figure S1). Consequently, we isolated **1** and **2** by preparative HPLC.

### 2.2. Structure Elucidation

**Figure 2:**
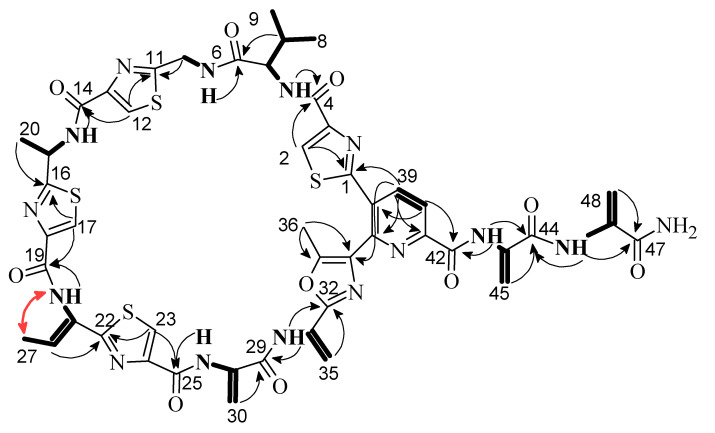
Selected ^1^H,^1^H COSY (bold lines), ^1^H,^13^C HMBC (black arrows) and ^1^H,^1^H NOESY (red arrow) correlations of **1.**

Litoralimycin A (**1**) was isolated as a light-yellow oil. The molecular formula of C_48_H_45_N_15_O_10_S_4_ was derived from its high-resolution electrospray ionization mass spectrometry (HRESIMS) peak observed at *m/z* 1120.2429. ^1^H and heteronuclear single-quantum correlation spectroscopy (HSQC) NMR spectra indicated the presence of 5 methyls, 4 exomethylenes and 1 low-field aliphatic methylene, and 7 olefinic/aromatic as well as 3 aliphatic methines, in addition to 10 exchangeable protons bound to heteroatoms (Table 1). The number of exomethylenes in combination with the four sulfur atoms gave an early hint towards a thiopeptide. The ^13^C spectrum indicated the presence of 9 additional carbonyls as well as 19 further olefinic carbons bearing no hydrogens. Based on correlation spectroscopy (COSY), total correlation spectroscopy (TOCSY) and heteronuclear multiple-bond correlation spectroscopy (HMBC) correlations, the planar thiopeptide structure, containing four thiazole (Thz), a valine (Val), an oxazole (Oxa), a pyridine (Pyr) and three dehydroalanine (Dala) units, was established (Figure 2). The rotating frame nuclear Overhauser effect spectroscopy (ROESY) correlation between H_3_-27 and 21-NH established the Δ^21,26^ double bond geometry as *Z*. Since thiazole amino acids racemize very easily during acid hydrolosis, the configuration of C-15 was determined by ozonolysis of the aromatic ring for preservation of the chiral center followed by acid hydrolosis [6]. After ozonolysis, hydrolyzation and derivatization with FDAA, we detected l-Val and l-Ala according to Marfey’s method [7]. Thus, both C-5 and C-15 are S-configured.

HRESIMS data of **2** gave a molecular formula of C_45_H_42_N_14_O_9_S_4_, implying the formal loss of a C_3_H_3_NO fragment compared to **1**. The proton and carbon NMR spectra of **2** were highly similar to those of **1**, with the key difference of the absence of a terminal dehydroalanin moiety. Therefore, the structure of **2** was established as identical to that of **1** with its side chain truncated by one dehydroalanin moiety.

### 2.3. Bioactivity

Litoralimycins A (**1**) and B (**2**) were evaluated for their minimum inhibitory concentration (MIC). Both compounds showed only a very weak activity against *Staphylococcus aureus* Newman and *Bacillus subtilis* DSM10^T^ with MIC values of 66.7μg/mL, respectively. No further effects were detected against any Gram-negative bacteria or fungi. (Table S1). However, cytotoxic effects against different cell lines were detected for **1** (Table 2). Litoralimycin B (**2**) with its truncated side chain showed much weaker cytotoxic activity.

## 3. Discussion

Thiopeptides, or thiazolyl peptides, are highly modified sulfur-rich peptides of ribosomal origin. Over 100 chemical entities have been isolated in the last 50 years [9]. Of these entities, thiostrepton has been used as an FDA-approved active pharmaceutical ingredient for animals, and nosiheptide has been widely applied in veterinary antibiotics and food preservation. Their most characteristic feature is the central nitrogen-containing six-membered ring structure. Depending on the oxidation state of this central ring, thiopeptides can be classified into five different series [10]. The litoralimycins belong to the *d* series, which is the most numerous subgroup, due to their trisubstituted pyridine moiety. Another option to group the thiopeptides is based on the ring size of the main macrocycle, since ring sizes of 26, 29, 32 and 35 atoms are found. Specifically, litoralimycins **1** and **2** belong to a small family of compounds with an oxazolyl-thiazolyl-pyridine fragment embedded in a 35-membered (13-residue) peptidyl macrocycle [11]. Other members of this family, which have highest structural similarity to **1** and **2**, are the berninamycins, sulfomycin, thioplabin and TP-1161A. Common variations between members of this family are the exchange of thiazole by oxazole moieties, different methylation patterns and the size of the side chain (see Figure S3).

Most characterized thiopeptides display nanomolar potency toward Gram-positive bacteria by blocking protein translation, including the notorious pathogens methicillin-resistant *Staphylococcus aureus* (MRSA), vancomycin-resistant enterococci (VRE), and penicillin-resistant *Streptococcus pneumoniae* (PRSP). Their mechanism of action, acting as protein synthesis inhibitors, correlates with the size of the primary macrocycle: 29-atom macrocycles bind to elongation factor EF-TU, while 26- and 32-atom macrocycles bind to the interface of protein L11 and the 23S rRNA within the 50S ribosomal subunit. The general molecular target of compounds with the largest 35-membered macrocycle remains unknown [10], although berninamycin was reported to target the 50S ribosome, in a similar manner to thiostrepton [11].

Using NMR and biochemical assays, a three-dimensional interaction model was developed, identifying l-Thr as a preserved region for the interaction with the ribosome/L11 complex [12]. This residue is missing for the litoralimycins, since **1** and **2** bear a l-Val at this position instead. In conformity with **1** and **2**, radamycin also has a mutated residue with l-Val replacing l-Thr. In analogy to the litoralimycins, radamycin was devoid of any antibacterial activity in agar diffusion assays [13,14]. Nevertheless, radamycin showed a strong *tipA* promoting activity. *tipA* gene promotion, encoding the two thiostrepton-induced proteins (Tip) TipAL and TipAS. The latter serves as a defense mechanism for bacteria against thiopeptides. Since the *tipA* promotion activity was identified to be dependent on a dehydroalanine-containing tail close to the six-membered central scaffold [15], we expect the litoralimycins to be *tipA* activators.

Besides the aforementioned effects on bacteria, some thiopeptides show good anticancer activities [9]. For thiostrepton it was shown that this activity is based on its effect of reducing transcriptional activity of the forkhead box M1 (FOXM1). FOXM1 is an oncogenic transcription factor that is upregulated in a wide range of cancers. It is involved in the regulation of the cell cycle and promotes angiogenesis, as well as metastasis. Because treatment with thiostrepton had an effect on cell proliferation and cell-cycle progression in MCF-7 cells [16], and litoralimycins A (**1**) was strongly active against cell line MCF-7 in our test assay, FOXM1 might be the molecular target in common.

## 4. Materials and Methods

### 4.1. General

HRESIMS mass spectra were measured with an Agilent 1200 series HPLC–UV system in combination with an ESI-TOF-MS (Maxis, Bruker) (column 2.1 × 50 mm, 1.7 µm, C18 Acquity UPLC BEH (Waters), solvent A: H_2_O + 0.1% formic acid, solvent B: ACN + 0.1% formic acid, gradient: 5% B for 0.5 min increasing to 100% B in 19.5 min, maintaining 100% B for another 5 min, RF = 0.6 mL min^−1^, UV detection 200–600 nm). NMR spectra were recorded on a Bruker Avance III 700 MHz spectrometer with a 5 mm TCI cryoprobe (^1^H 700 MHz, ^13^C 175 MHz, ^15^N 71 MHz). Chemical shifts *δ* were referenced to DMSO-*d*_6_ (^1^H, *δ* = 2.50 ppm; ^13^C, *δ* = 39.51 ppm). UV spectra were recorded using the Shimadzu UV*vis* spectrophotometer UV-2450. Optical rotation was determined using a PerkinElmer 241 polarimeter. Preparative isolation of the major components was achieved with preparative HPLC-system Gilson PLc 2250 (C18 column-nucleodur-7 µm-125 × 40 mm-RP 100, using Solvent A: H_2_O, solvent B: acetonitrile, gradient system: 20% B for 0.5 min increasing to 50% B in 30 min, 50% B to 100% B for 20 min, maintaining 100% B for 5 min, flow rate = 50 mL/min, detection at 200–600 nm).

### 4.2. Strain Maintenance

*Streptomonospora* sp. DSM 106425T was isolated in 2017 by the serial dilution method from a sand sample that had been collected from a beach of the North Sea at Cuxhaven, Germany. The strain grew well in the presence of 7% NaCl. This percentage of sodium chloride was added to all media that we used for culturing and production media. A section of the agar containing bacterial colonies and aerial mycelium was stored in glycerol 20% at −80 °C. The strain was transferred to 100 mL of liquid GYM medium (0.4% glucose, 0.4% yeast extract, 1% malt extract, 0.2% CaCO_3_; pH 7.2). The inoculated flask was incubated on a rotary shaker (160 rpm) for 5 days at 30 °C.

### 4.3. Fermentation, Extraction, and Isolation of Compounds

The 5-day-old preculture was transferred to production medium 1:10 in eight 1000-mL flasks, filled with 800 mL of medium 5294 (1% soluble starch, 0.2% yeast extract, 1% glucose, 1% glycerol, 0.25% corn steep liquor, 0.2% peptone, 0.1% NaCl, 0.3% CaCO_3_; pH 7.2) incubated at 30 °C for 8 days on rotary shaker (160 rpm). The 8-day-old culture medium was centrifuged using a Sorvall RC-5 refrigerated superspeed centrifuge for 30 min, at 8500 rpm. The supernatant was discarded and 311 g cell mass was extracted with two liters of acetone three time in an ultrasonic bath (3 × 30 min). The solution obtained was evaporated to yield an aqueous phase, which was further extracted with ethyl acetate (3 × 500 mL) and the ethyl acetate portion was dried out with evaporator to yield 575.2 mg of crude cell mass extract. An initial pre-separation of 575.2 mg crude extract was applied with a Strata TM-X 33 UM Polymeric reversed phase 1 g/12 mL Giga tube (Phenomenex) and washed three times with methanol. Subsequently, fractionation of 265.2 mg crude extract was completed by preparative HPLC (Gilson) (1 run using a linear gradient of solvent B from 20% to 50% solvent B in 30 min, 50% to 100% B in 20 min followed by isocratic conditions for 10 min at a flow rate of 50 mL/min). Fractions were collected and combined according to UV absorption at 220, 280 and 350 nm and yielded 5.6 mg of **1** at a retention time of 37.5–38.5 min and 0.5 mg of **2** at 36.5–37 min, respectively.

Litoralimycin A (**1**): light-yellow oil; [α]_D_ = +251 (*c* = 1 mg/mL in acetone); ^1^H NMR (700 MHz, DMSO-*d*_6_): see Table 1; ^13^C NMR (175 MHz, DMSO-*d*_6_): see Table 1; ESI-MS: *m/z* 1120.38 [M + H]^+^, 1118.43 [M + H]^+^; HRESIMS: *m*/*z* 1120.2429 [M + H]^+^ (calcd. for C_48_H_46_N_15_O_10_S_4_ 1120.2429), 1142.2246 [M + Na]^+^ (calcd. for C_48_H_45_N_15_O_10_S_4_Na 1142.2249).

Litoralimycin B (**2**): light-yellow oil; [α]_D_ = +399 (*c* = 1 mg/mL in acetone); ^1^H NMR (700 MHz, DMSO-*d*_6_): *δ*_H_ 10.67 (br s, 43–NH), 9.81 (br s, 21–NH), 9.74 (br s, 31–NH), 9.69 (br s, 28–NH), 8.95 (dd, *J* = 6.2, 5.2 Hz, 10–NH), 8.75 (br d, *J* = 8.2 Hz, 15–NH), 8.52 (d, *J* = 8.2 Hz, 39–H), 8.45 (s, 2–H), 8.31 (s, 23–H), 8.29 (s, 17–H), 8.25 (s, 12–H), 8.22 (d, *J* = 8.2 Hz, 40–H), 8.17 (br s, 44–NH_a_), 8.07 (br d, *J* = 9.2 Hz, 5–NH), 7.66 (br s, 44–NH_b_), 6.58 (s, 45–H_a_), 6.52 (q, *J* = 6.9 Hz, 26–H), 6.48 (br s, 30–H_a_), 5.81 (br s, 45–H_b_), 5.72 (br s, 30–H_b_), 5.63 (br s, 35–H_a_), 5.44 (m, 15–H), 5.42 (br s, 35–H_b_), 4.70 (dd, *J* = 15.9, 6.2 Hz, 10–H_a_), 4.54 (dd, *J* = 15.9, 5.2 Hz, 10–H_b_), 4.37 (dd, *J* = 9.2, 7.3 Hz, 5–H), 2.64 (s, 36–H_3_), 2.10 (dspt, *J* = 7.2, 6.9 Hz, 7–H), 1.79 (br d, *J* = 6.9 Hz, 27–H_3_), 1.55 (br d, *J* = 6.9 Hz, 20–H_3_), 0.90 (d, *J* = 6.9 Hz, 9–H_3_), 0.87 (d, *J* = 6.9 Hz, 8–H_3_) ppm; ^13^C NMR (175 MHz, DMSO-*d*_6_): *δ*_C_ 173.6 (C, C–16), 170.9 (C, C–6), 168.6 (C, C–11), 167.3 (C, C–22), 164.9 (C, C–44), 163.8 (C, C–1), 161.2 (C, C–42), 159.98 (C, C–4), 159.94 (C, C–14), 159.2 (C, C–19), 158.9 (C, C–25), 155.6 (C, C–32), 150.2 (C, C–33), 149.22 (C, C–3), 149.18 (C, C–41), 148.7 (C, C–18), 148.6 (2xC, C–13, C–24), 147.7 (C, C–37), 141.2 (CH, C–39), 133.8 (C, C–28), 133.6 (C, C–43), 133.1 (C, C–34), 130.6 (C, C–38), 129.2 (C, C–21), 129.0 (C, C–31), 128.3 (CH, C–26), 125.3 (CH, C–12), 125.1 (CH, C–17), 125.0 (CH, C–23), 104.5 (CH_2_, C–30), 102.5 (CH_2_, C–45), 58.2 (CH, C–5), 46.9 (CH, C–15), 40.0 (CH_2_, C–10), 30.7 (CH, C–7), 20.6 (CH_3_, C–20), 19.3 (CH_3_, C–8), 18.4 (CH_3_, C–9), 14.2 (CH_3_, C–27), 11.6 (CH_3_, C–26); ESI-MS: *m/z* 1051.32 [M + H]^+^, 1049.34 [M + H]^+^; HRESIMS: *m*/*z* 1051.2219 [M + H]^+^ (calcd. for C_45_H_43_N_14_O_9_S_4_ 1051.2215), 1073.2031 [M + Na]^+^ (calcd. for C_45_H_42_N_14_O_9_S_4_Na 1073.2034).

### 4.4. Ozonolysis, Hydrolysis and Marfey’s Derivatization with l-FDAA

For the ozonolysis reaction a stream of O_3_ was bubbled through a solution of **1** (2.3 mg) dissolved in methanol (6 mL) at −78 °C until the solution obtained a characteristic blue color and stirred for 30 min. Subsequently, the solvent was removed in vacuo and the resulting oxidized material was subjected to hydrolysis in 3 mL of 6 n HCl at 110 °C for 24 h as described in [6]. Afterwards, the solvent was removed under a stream of nitrogen for 3 h and the remainder dissolved in H_2_O (200 μL), of which 100 μL were proceeded further. A total of 1 n NaHCO_3_ (20 μL) and 1% 1-fluoro-2,4-dinitrophenyl-5-Lalaninamide (100 μL in acetone) were added, and the mixture was heated at 40 °C for 40 min [17]. After being cooled to room temperature, the solutions were neutralized with 2 n HCl (20 μL) and evaporated to dryness. The residues were dissolved in MeOH and analyzed by HPLC−MS. Retention times in minutes of FDAA-derivatized amino acids were 6.5 for Val and 5.0 for Ala. Retention times of the authentic amino acid standards were l-Val 6.5, dl-Val 6.5/7.5, l-Ala 5.0, dl-Ala 5.0/6.0.

### 4.5. Minimum Inhibitory Concentrations

Minimum Inhibitory Concentrations (MIC) were investigated in a serial dilution assay in 96-well microtiter plates in YM medium for yeasts and filamentous fungi and BD^TM^ Difco^TM^ Müller-Hinton Broth for bacteria, as previously published [18].

### 4.6. Cytotoxicity Assay

The in vitro cytotoxicity assay was carried out as described earlier [8].

### 4.7. HPLC Fractionation and Bioassays in 96-Well Plates

An Agilent 1260 Series HPLC–UV system equipped with a Waters, XBridge BEHC18, 2.1 mm 100 mm column (pore size 135 Å, particle size 3.5 µm, solvent A: H_2_O-acetonitrile (95/5), 5 mmol NH_4_Ac, 0.04 mL/L CH_3_COOH; solvent B: H_2_O-acetonitrile (5/95), 5 mmol NH_4_Ac, 0.04 mL/L CH_3_COOH; gradient system: 10% B increasing to 100% B in 30 min; flow rate 0.3 mL/min; 40 °C; UV-detection at 210–450 nm) was used for the chromatographic fractionation of crude extracts. The same HPLC gradient was used as for the high-resolution electrospray ionization mass spectrometry (HRESIMS) instrument. The flow-through was collected in 30 s intervals into a 96-well microtiter plate. Afterwards, the plates were dried by a constant nitrogen-flush for 40 min, inoculated with 150 mL indicator bacteria per well and incubated as described [17]. After 24 h the plates were evaluated and documented employing a custom-made mirror stand and a CANON EOS 60D digital camera.

## 5. Conclusions

Two new thiopeptide antibiotics were isolated from a new actinomycetes bacterium, which was isolated from a sand sample collected at a Wadden Sea beach. While their planar structures were elucidated by NMR and MS data, their absolute configuration was determined by degradation by ozonolysis and hydrolosis followed by Marfey’s method. Their spectrum of biological activities is rare, because they are cytotoxic but possess virtually no antibacterial activities.

## Figures and Tables

**Table 1:** NMR data (^1^H 700 MHz, ^13^C 175 MHZ) of **1** in DMSO-*d*_6_.

Unit	Pos	*δ*_C_	*δ*_H_	Unit	Pos	*δ*_C_	*δ*_H_
Thz 1	1	163.6, C		Dala 1	28	133.7, C	
	2	127.1, CH	8.47, s		28NH	NH	9.70, br s
	3	149.3, C			29	162.5, C	
	4	159.9, C			30	104.5, CH_2_	5.74, br s
Val	5	58.2, CH	4.38, dd (9.3, 7.4)		30		6.49, br s
	5NH	NH	8.08, d (9.3)	Oxa	31	129.0, C	
	6	170.9, C			31NH	NH	9.78, br s
	5	30.6, CH	2.11, m		32	155.8, C	
	8	19.3, CH_3_	0.91, d (6.7)		33	150.0, C	
	9	18.4, CH_3_	0.88, d (6.7)		34	133.1, C	
Thz 2	10	40.2, CH_2_	4.55, m		35	109.5, CH_2_	5.64, br s
			4.70, dd (16.0,6.4)				5.43, br s
	10NH	NH	8.95, t (6.4)		36	11.4, CH_3_	2.60, s
	11	168.6, C		Pyr	37	147.6, C	
	12	125.3, CH	8.25, s		38	130.8, C	
	13	148.6, C			39	141.1, CH	8.60, d (8.1)
	14	159.9, C			40	121.1, CH	8.25, d (8.1)
Thz 3	15	46.8, CH	5.44, m		41	149.0, C	
	15NH	NH	8.74, d (8.2)		42	161.4, C	
	16	173.5, C		Dala 2	43	134.0, C	
	17	125.1, CH	8.29, m		43NH	NH	10.5, br s
	18	148.7, C			44	161.9, C	
	19	159.2, C			45	104.0, CH_2_	5.82, br s
	20	20.5, CH_3_	1.55, d (6.9)				6.61, br s
Thz 4	21	129.2, C		Dala 3	46	135.3, C	
	21NH	NH	9.80, br s		46NH	NH	9.54, br s
	22	167.3, C			47	165.1, C	
	23	125.0, CH	8.31, s		47NH	NH_2_	7.48, br s
	24	148.5, C				NH_2_	7.91, br s
	25	158.8, C			48	106.7, CH_2_	5.96, s
	26	128.3, CH	6.52, d (6.9)				5.67, s
	27	14.1, CH_3_	1.79, d (6.9)				

**Table 2:** Cytotoxic activities of litoralimycins A (**1**) and B (**2**) against different cell lines. Values indicate IC_50_ in µg/mL.

Compound	L929	KB3.1	MCF-7	SKOV-3	A431	PC-3
**1**	2.9	2.6	1.0	28	0.8	31
**2**	24.0	/	n.t. ^1^	n.t.	n.t	n.t
**epothilon B [8]**	0.00082	0.000065	0.000048	0.000095	0.000045	0.0001

^1^ n.t.: not tested

## Supplementary Materials

The following are available online at https://www.mdpi.com/1660-3397/18/6/280/s1, Figure S1: Fractionation analysis of *Streptomonospora* sp. M2 crude extract. Table S1: Antimicrobial activity of **1** and **2**, Figure S2: Observed TOCSY, HMBC and ROESY correlations for **1**, Figure S3: Known compounds structurally related to litoralimycins, Figure S4: HPLC–MS and UV–Vis chromatograms of *Streptomonospora* sp. M2 and HRESIMS data, Figure S5: HPLC–ESIMS spectrum of litoralimycin A (**1**), Figure S6: ^1^H NMR spectrum (700 MHz, DMSO-*d*_6_) of litoralimycin A (**1**), Figure S7: ^13^C NMR spectrum (175 MHz, DMSO-*d*_6_) of litoralimycin A (**1**), Figure S8: COSY NMR spectrum (700 MHz, DMSO-*d*_6_) of litoralimycin A (**1**), Figure S9: ROESY NMR spectrum (700 MHz, DMSO-*d*_6_) of litoralimycin A (**1**), Figure S10: HSQC NMR spectrum (700 MHz, DMSO-*d*_6_) of litoralimycin A (**1**), Figure S11: HMBC NMR spectrum (700 MHz, DMSO-*d*_6_) of litoralimycin A (**1**), Figure S12: Marfey derivatization for determination of type and configuration of amino acids in litoralimycin A (**1**), Figure S13: HPLC-ESIMS spectrum of litoralimycin B (**2**), Figure S14: ^1^H NMR spectrum (700 MHz, DMSO-*d*_6_) of litoralimycin B (**2**), Figure S15: ^13^C NMR spectrum (175 MHz, DMSO-*d*_6_) of litoralimycin B (**2**), Figure S16: COSY NMR spectrum (700 MHz, DMSO-*d*_6_) of litoralimycin B (**2**), Figure S17: ROESY NMR spectrum (700 MHz, DMSO-*d*_6_) of litoralimycin B (**2**), Figure S18: HSQC NMR spectrum (700 MHz, DMSO-*d*_6_) of litoralimycin B (**2**), Figure S19: HMBC NMR spectrum (700 MHz, DMSO-*d*_6_) of litoralimycin B (**2**), Figure S20: MS/MS data for **1** and **2** with fragmentation of [M + Na]^+^ ions.
