# Supplementary material for: Litoralimycins A and B, New Cytotoxic Thiopeptides from Streptomonospora sp. M2
Source: Mar Drugs. 2020 May 26;18(6):280. doi: 10.3390/md18060280 (PMC7345755; doi:10.3390/md18060280)
Supplement: Supplementary file 1 [file marinedrugs-18-00280-s001.pdf]

Article

Litoralimycins A and B, new cytotoxic thiopeptides from *Streptomonospora* sp. M2

Shadi Khodamoradi [1,2], Marc Stadler [2,3], Joachim Wink [1,2,*] and Frank Surup [2,3,*]

1 Microbial strain collection, Helmholtz-Centre for Infection Research (HZI), Inhoffenstr. 7, 38124 Braunschweig, Germany; shadi.khodamoradi@helmholtz-hzi.de, joachim.wink@helmholtz-hzi.de.
2 German Centre for Infection Research (DZIF), partner site Hannover-Braunschweig, 38124 Braunschweig, Germany.
3 Microbial Drugs Department, Helmholtz-Centre for Infection Research (HZI), Inhoffenstr. 7, 38124 Braunschweig, Germany; marc.stadlerp@helmholtz-hzi.de, frank.surup@helmholtz-hzi.de.
* Correspondence: joachim.wink@helmholtz-hzi.de (biology), frank.surup@helmholtz-hzi.de (chemistry); Tel.: +49-351-6181-4223 (J.W.), +49-351-6181-4256 (F.S.)

------------------------Supporting Information------------------------

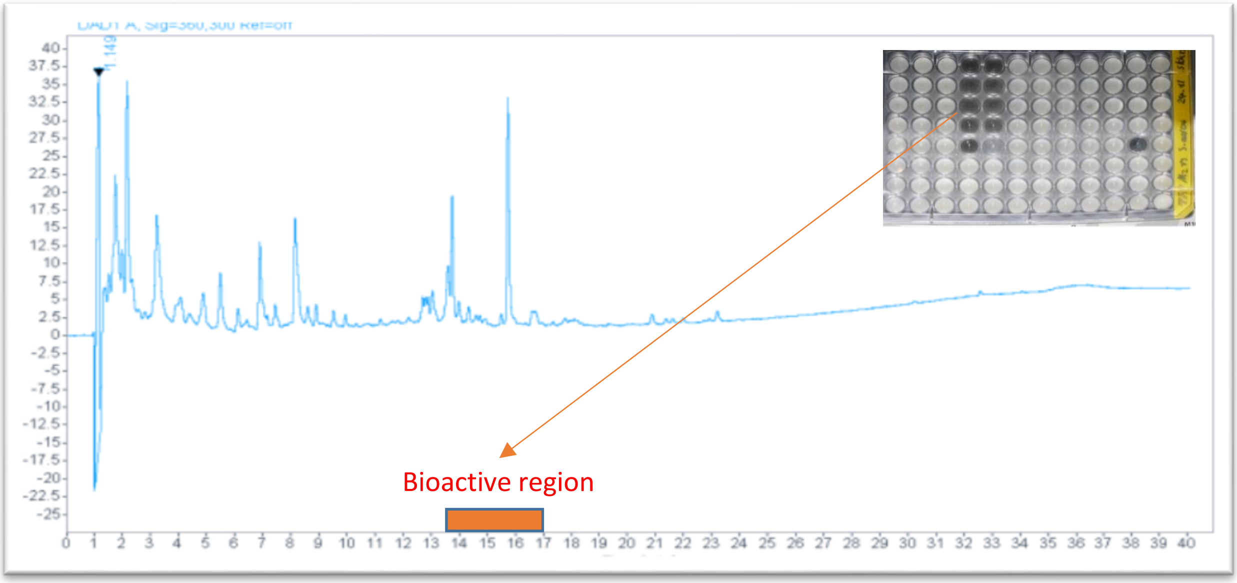

Figure S1: Fractionation analysis of *Streptomonospora* sp. M2 crude extract.

By fractionation of 5µL *Streptomonospora* sp. M2 crude extract (100x concentrated in methanol compared to culture volume) the reproducible growth inhibition of test organism *Staphylococcus aureus* was caused in wells D5 till E4, which had been collected from 13.5 to 17 minutes by HPLC fractionation.

Table S1. Antimicrobial activity of **1** and **2** versus different Gram-positive and Gram negative bacteria, fungal and yeast. n.i: no inhibition, T: Tetracycline, G: Gentamycin, N: Nystatin, K: Kanamycin

**Test organisms**	**compound 1**	**comound 2**	**Ref G-K-N-T**
Bacillus subtilis DSM10^T	66.7	66.7	16.6 T
Staphylococcus aureus Newman	66.7	66.7	1.02 G
Escherichia coli WT BW25113	n.i	n.i	1.02 G
Escherichia coli acrB JW0451-2	n.i	n.i	1.02 G
Pseudomonas aeruginosa PA14	n.i	n.i	1.2 G
Acinetobacter baumanni	n.i	n.i	1.2 G
Citrobacter freundii DSM 30039	n.i	n.i	1.02 G
Mycobact semegmatic ATCC700084	n.i	n.i	4.1 K
Mucor himalis DSM 2656 T	n.i	n.i	16.6 N
Candida albicans DSM1665	n.i	n.i	33.35 N
Pichia anomala DSM6766	n.i	n.i	33.35 N

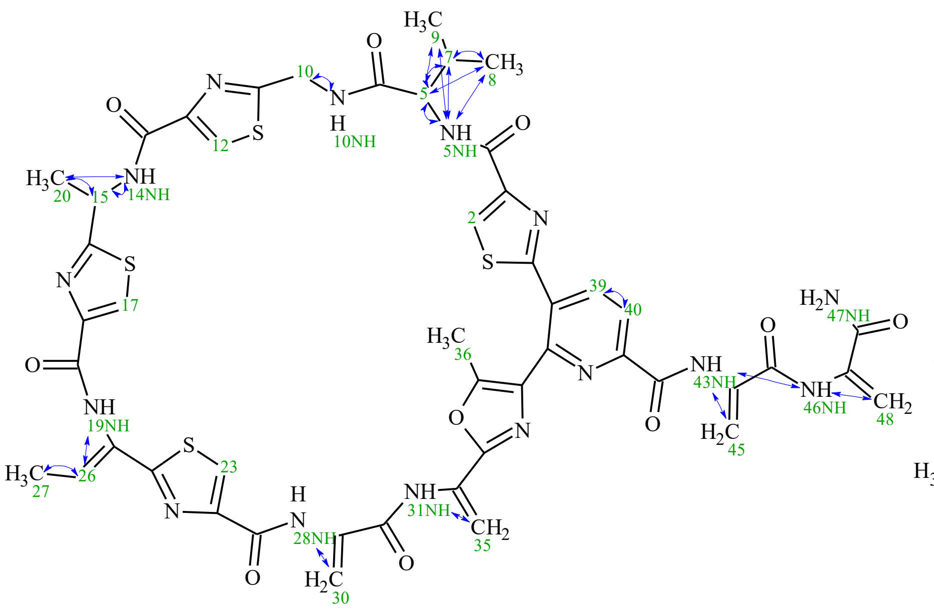

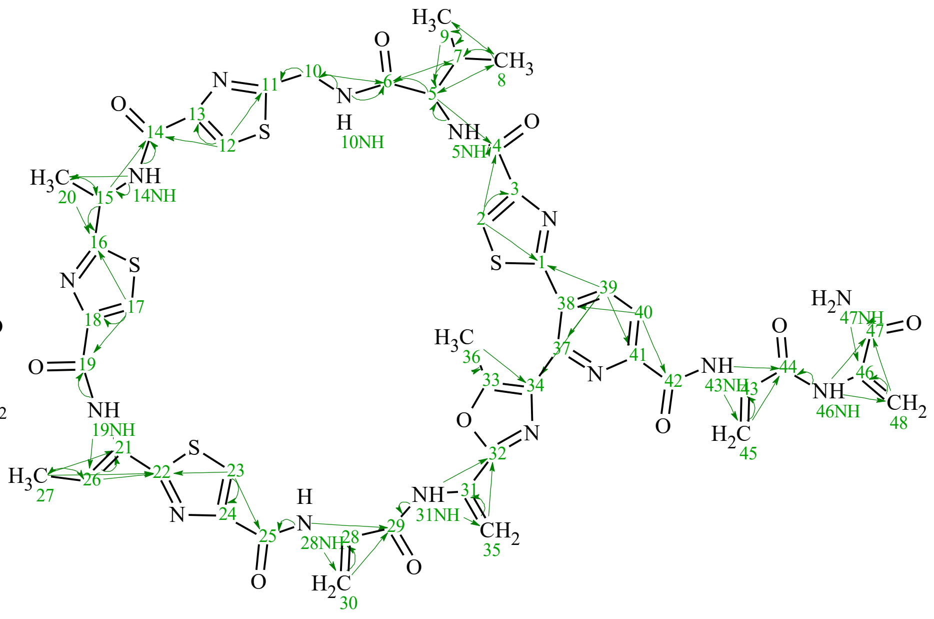

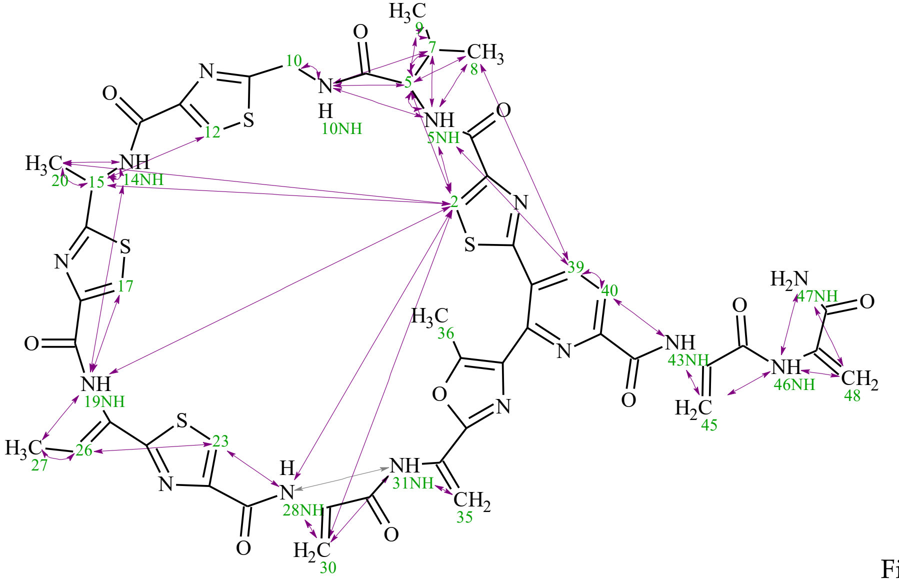

Figure S2. Observed a) TOCSY, b) HMBC and c) ROESY correlations for **1**.

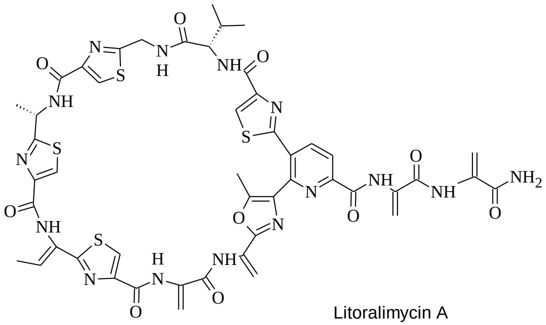

Litoralimycin A

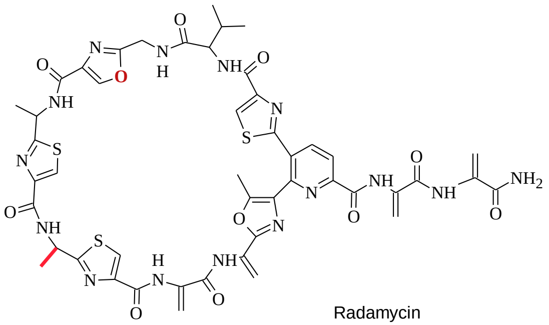

Radamycin

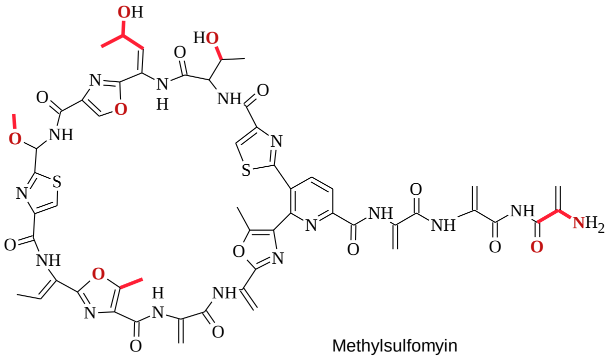

Methylsulfomyin

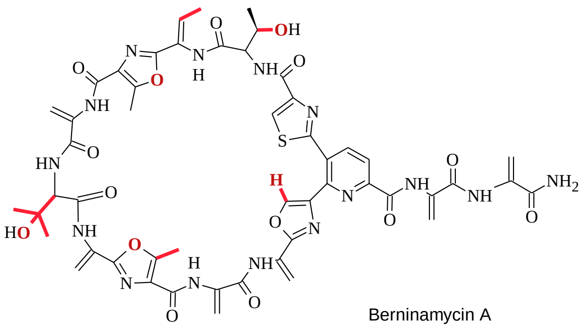

Berninamycin A

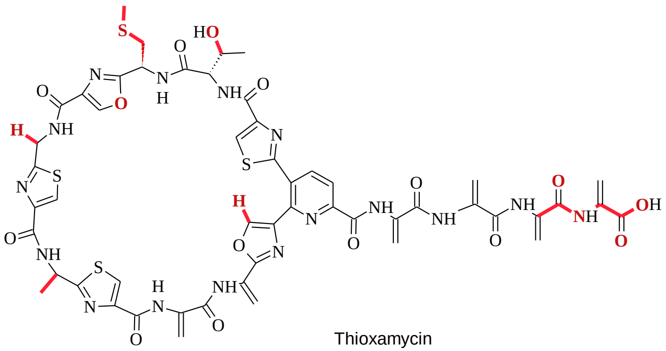

Thioxamycin

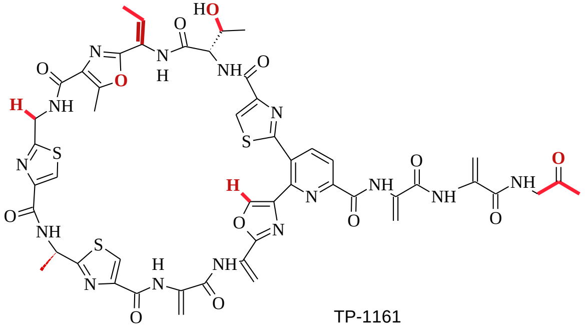

TP-1161

Figure S3. Known compounds structurally related to litoralimycins. Differences to **1** are highlighted in bold red.

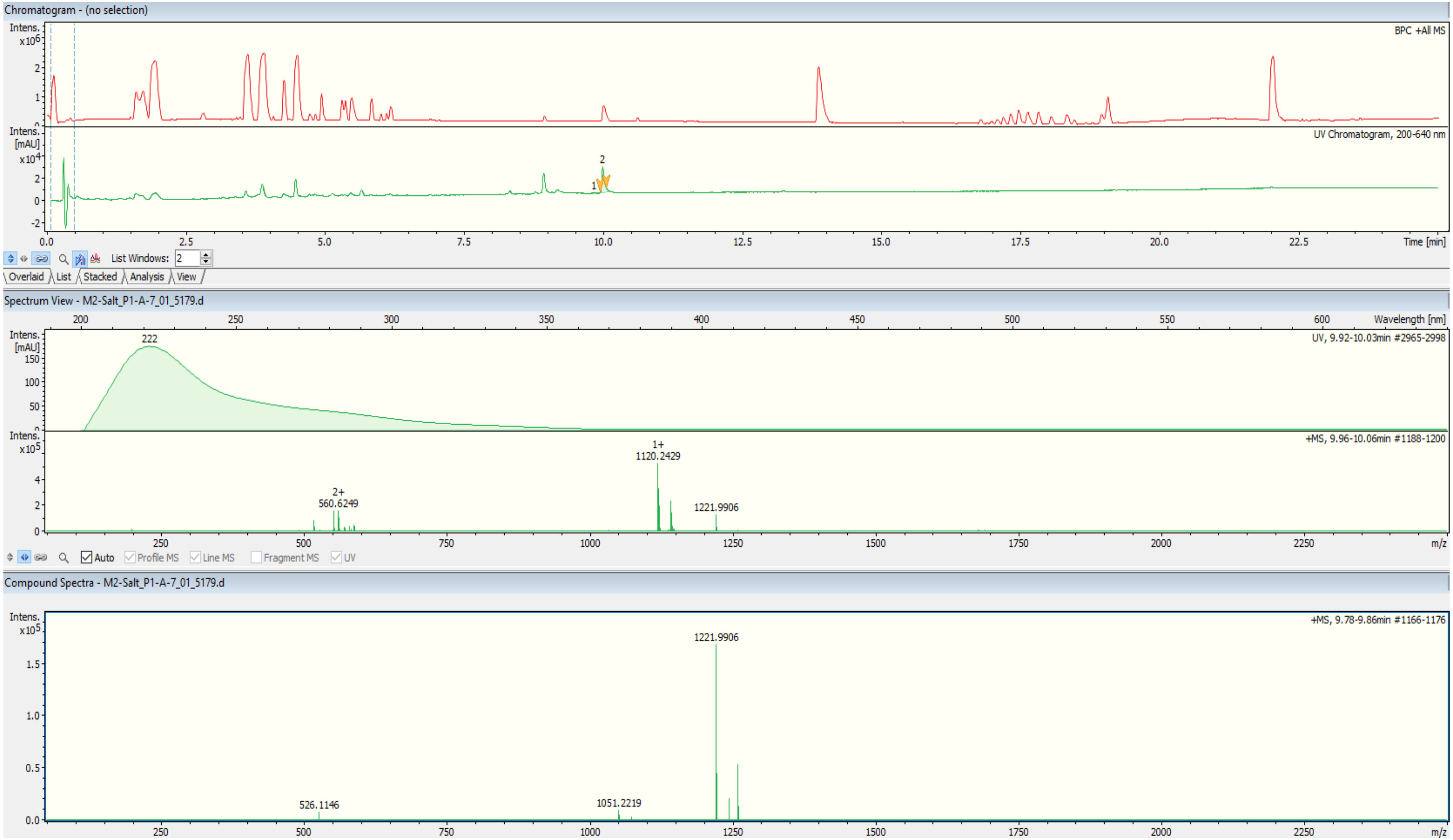

Figure S4: HPLC-MS (basepeak) and UV/Vis (200-640nm) chromatograms of a crude extract of *Streptomonospora* sp. M2 and HRESIMS data of litoralimycin A (**1**, middle) and B (**2**, bottom).

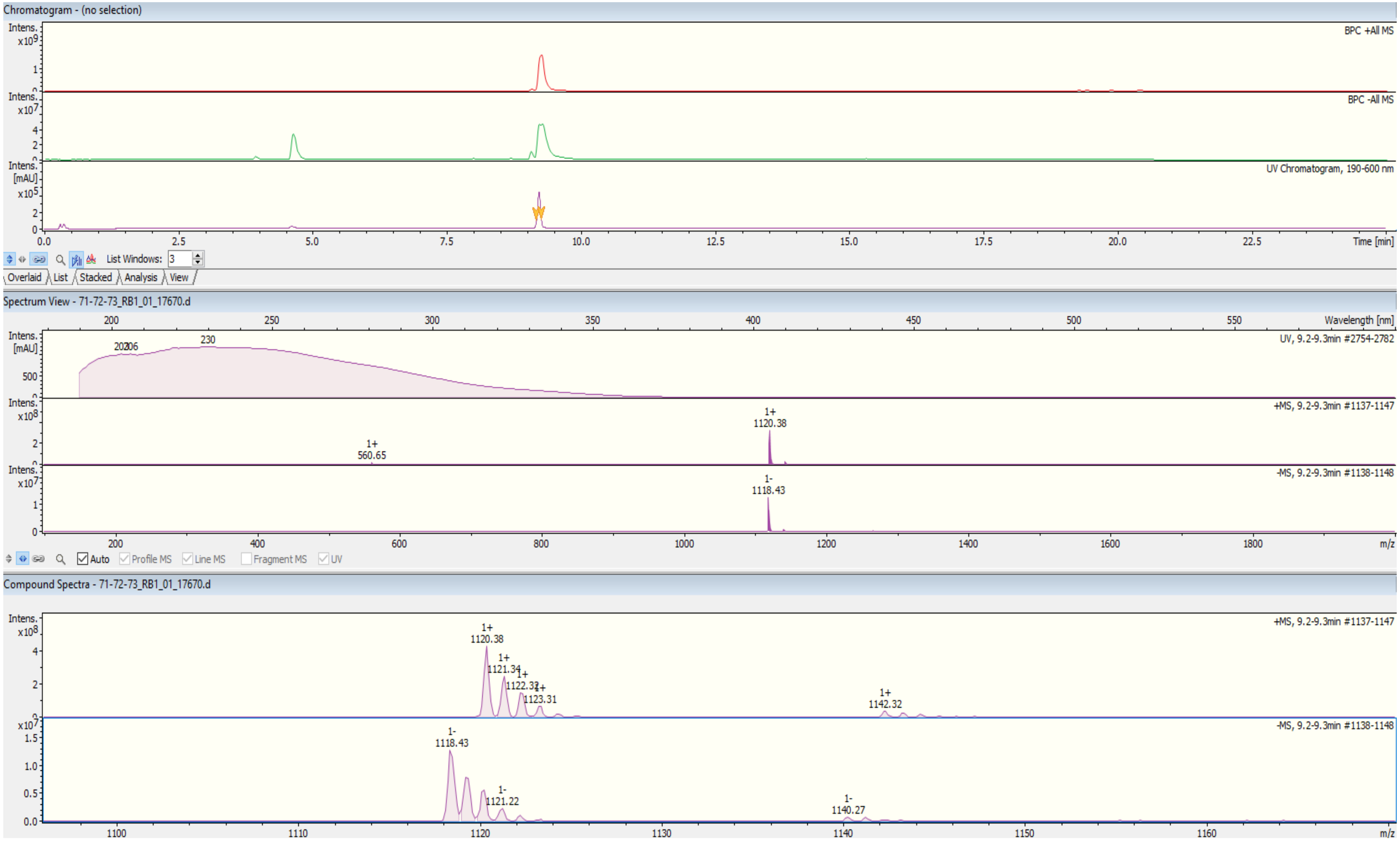

Figure S5: HPLC-ESIMS spectrum of litoralimycin A (**1**).

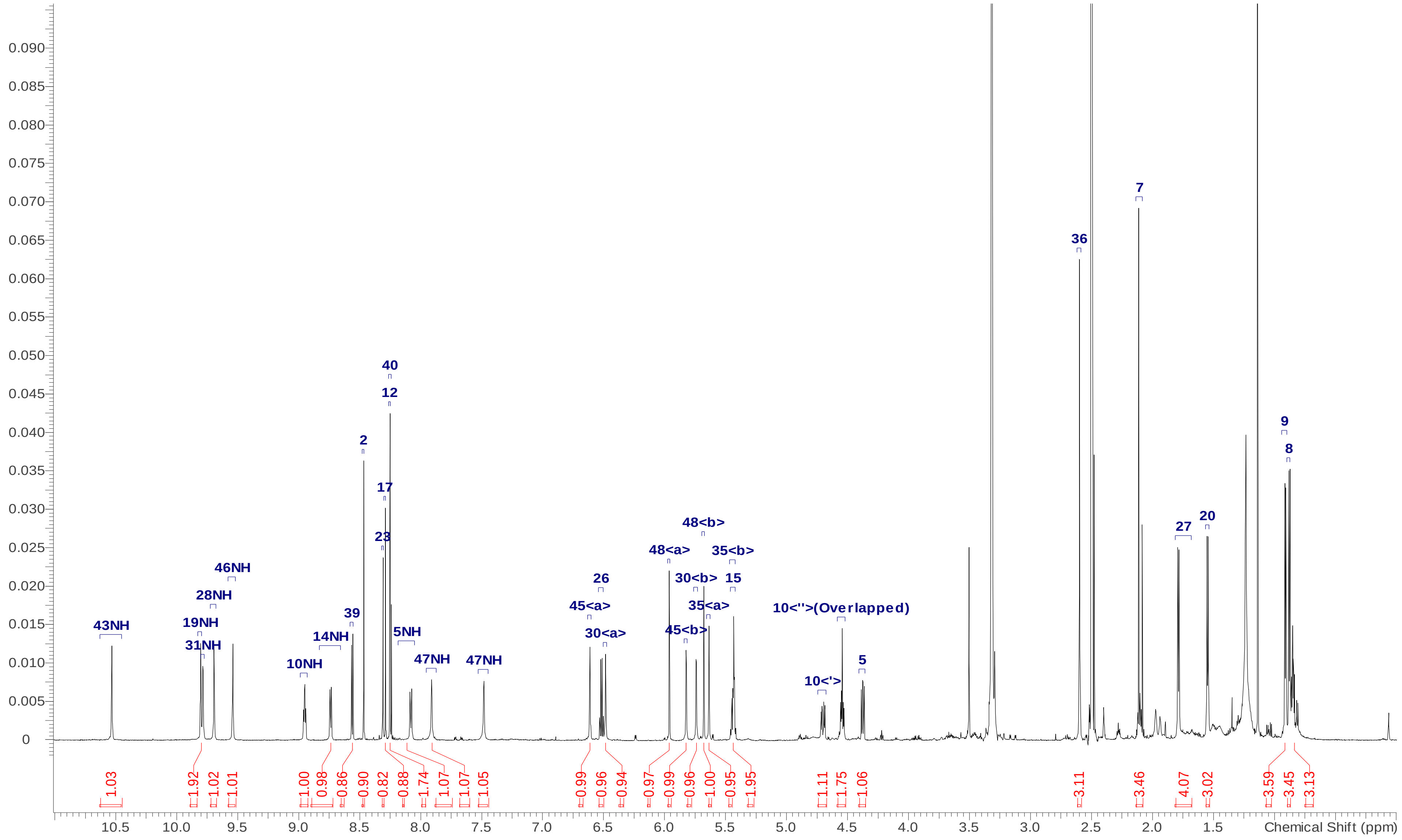

Figure S6: ^{1}H NMR spectrum (700 MHz, DMSO-d_6) of litoralimycin A (**1**).

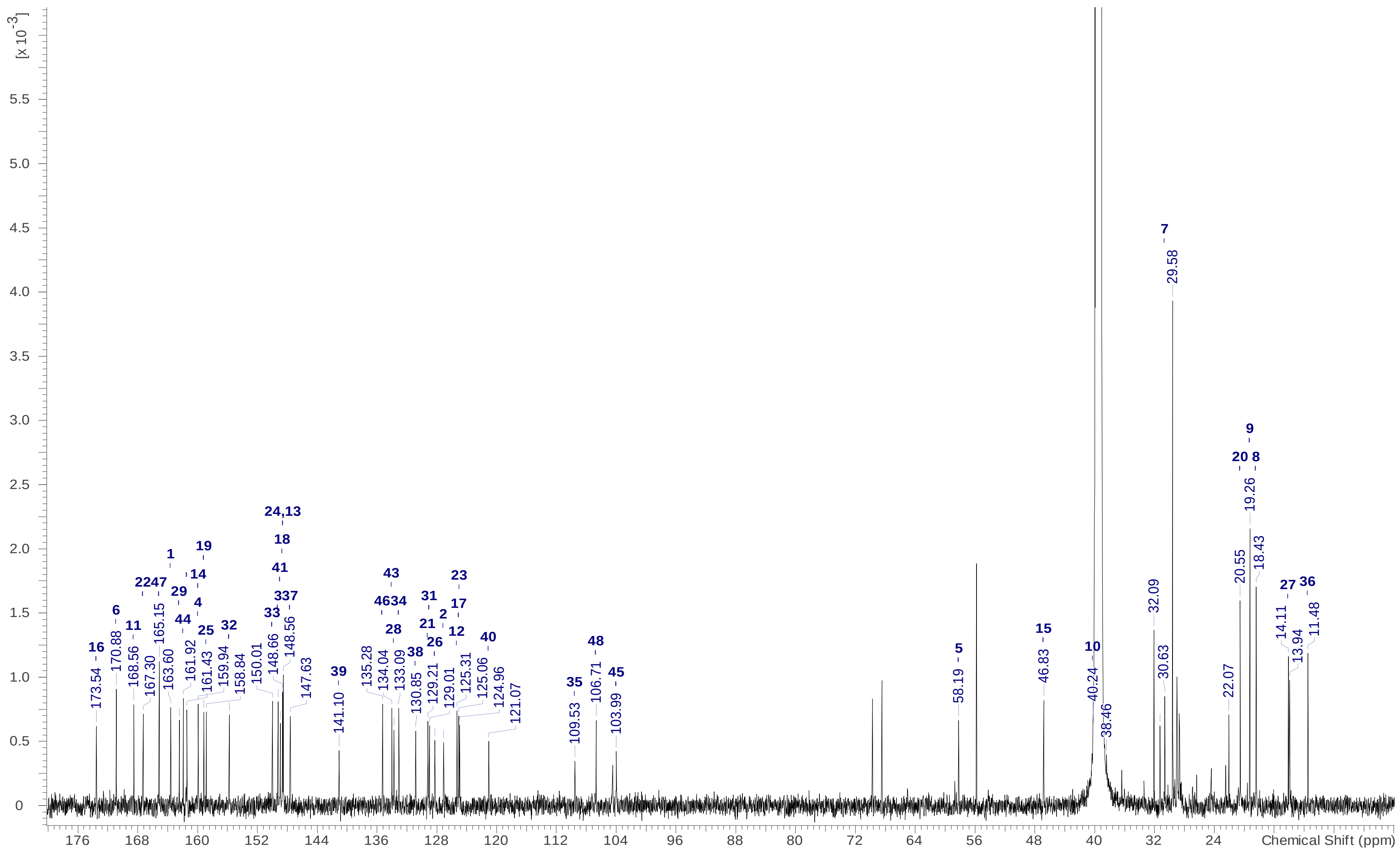

Figure S7: ^{13}C NMR spectrum (175 MHz, DMSO-d_6) of litoralimycin A (**1**).

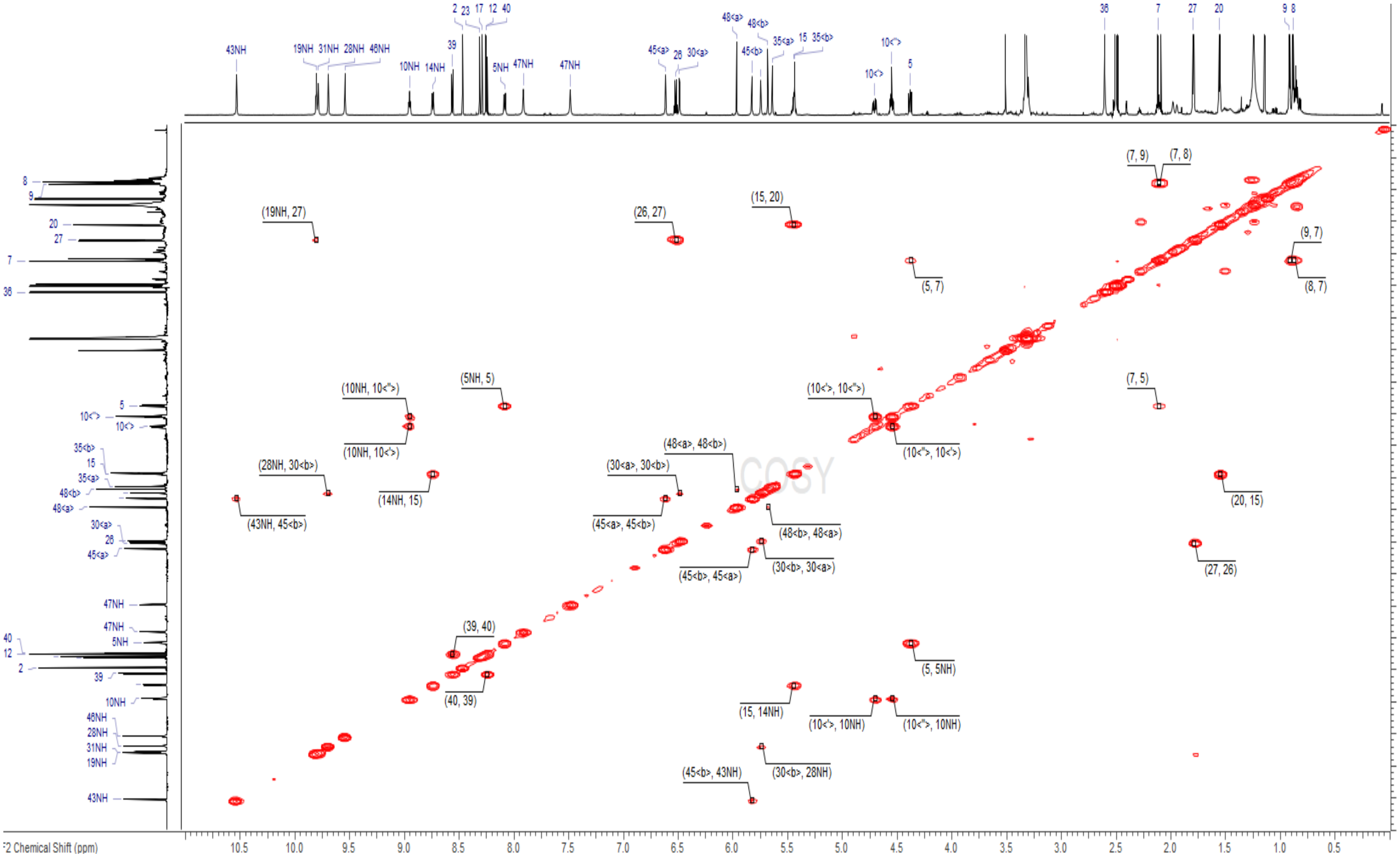

Figure S8: COSY NMR spectrum (700 MHz, DMSO-d_6) of litoralimycin A (**1**).

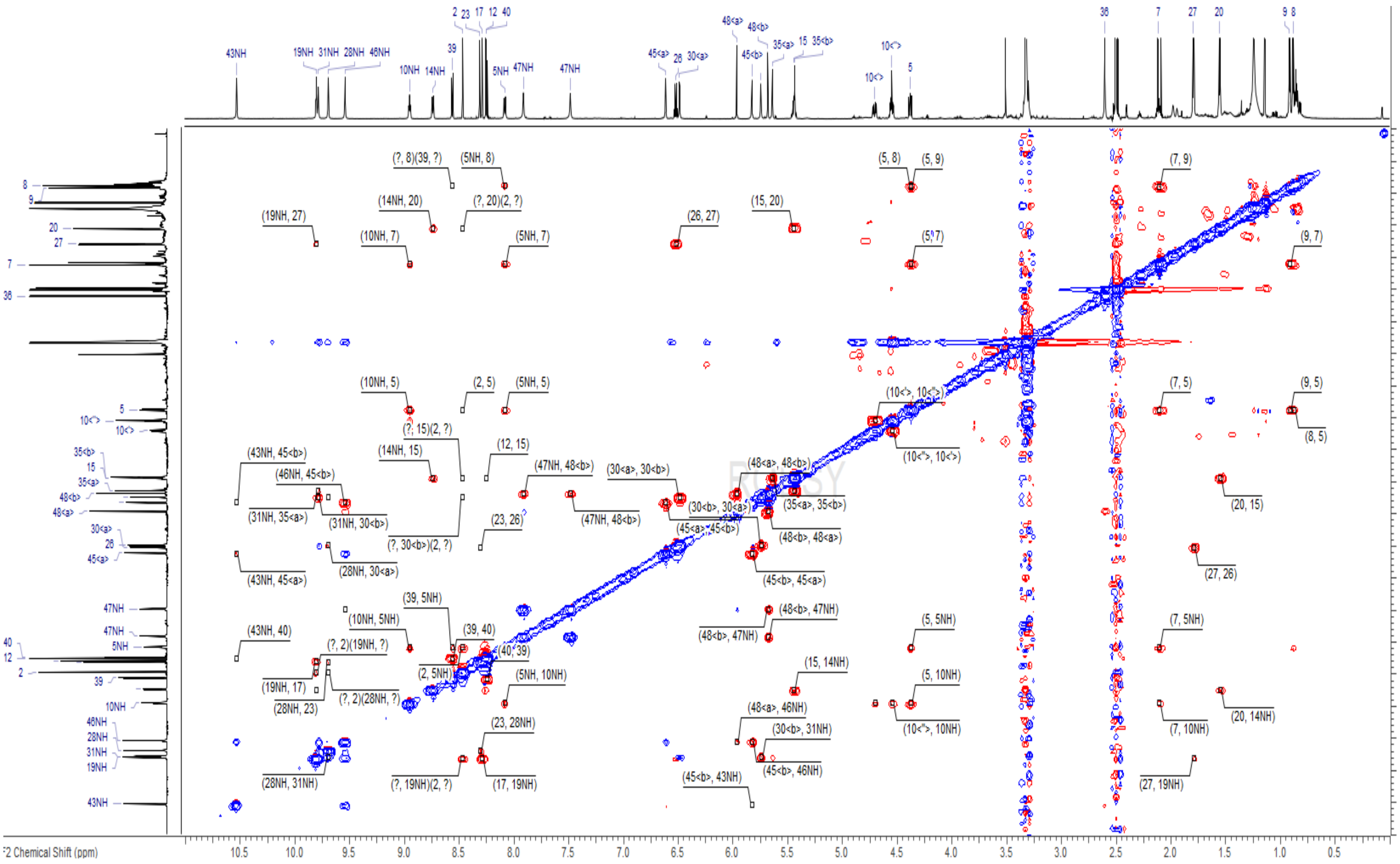

Figure S9: ROESY NMR spectrum (700 MHz, DMSO-d_6) of litoralimycin A (**1**).

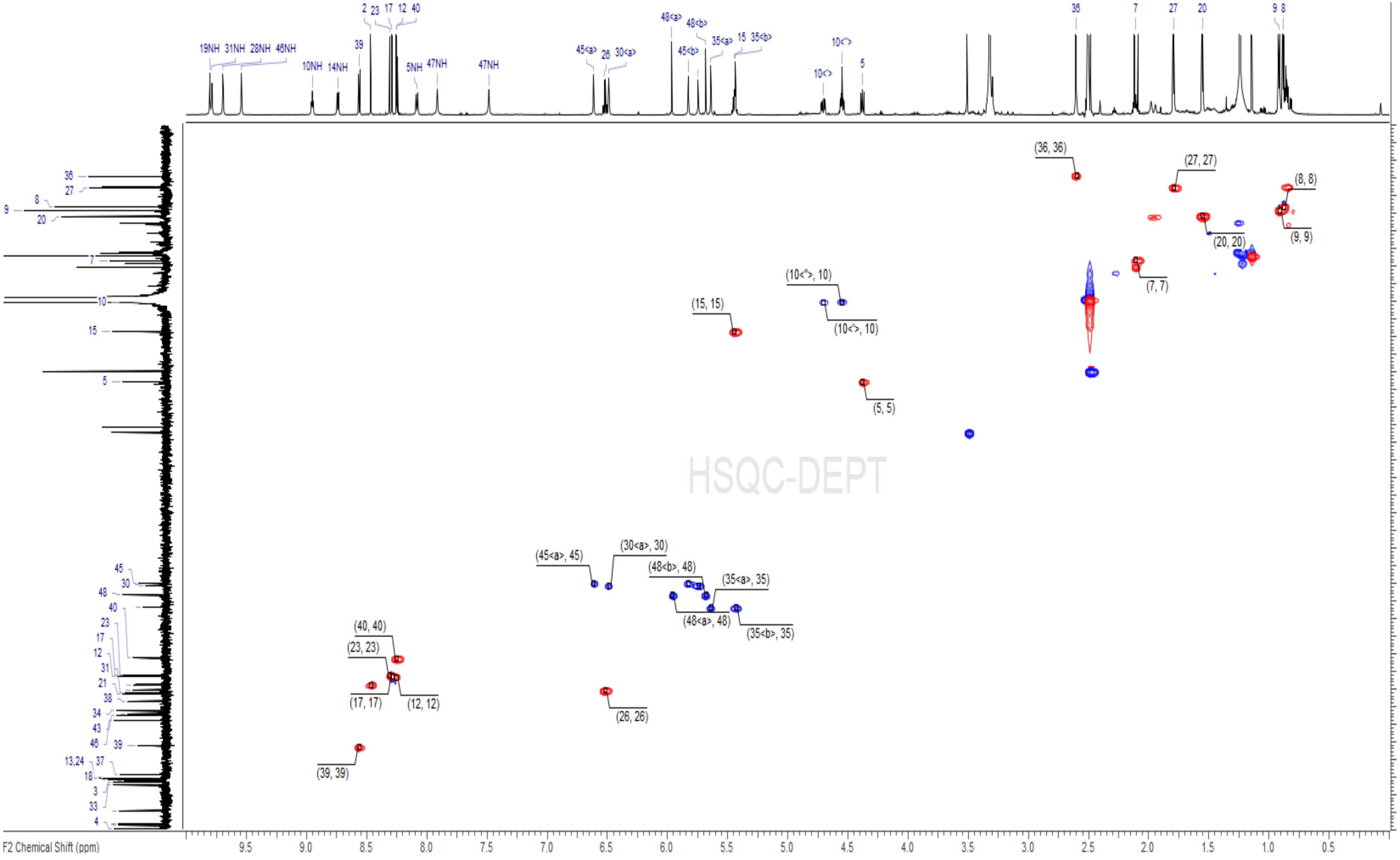

Figure S10: HSQC NMR spectrum (700 MHz, DMSO-d_6) of litoralimycin A (**1**).

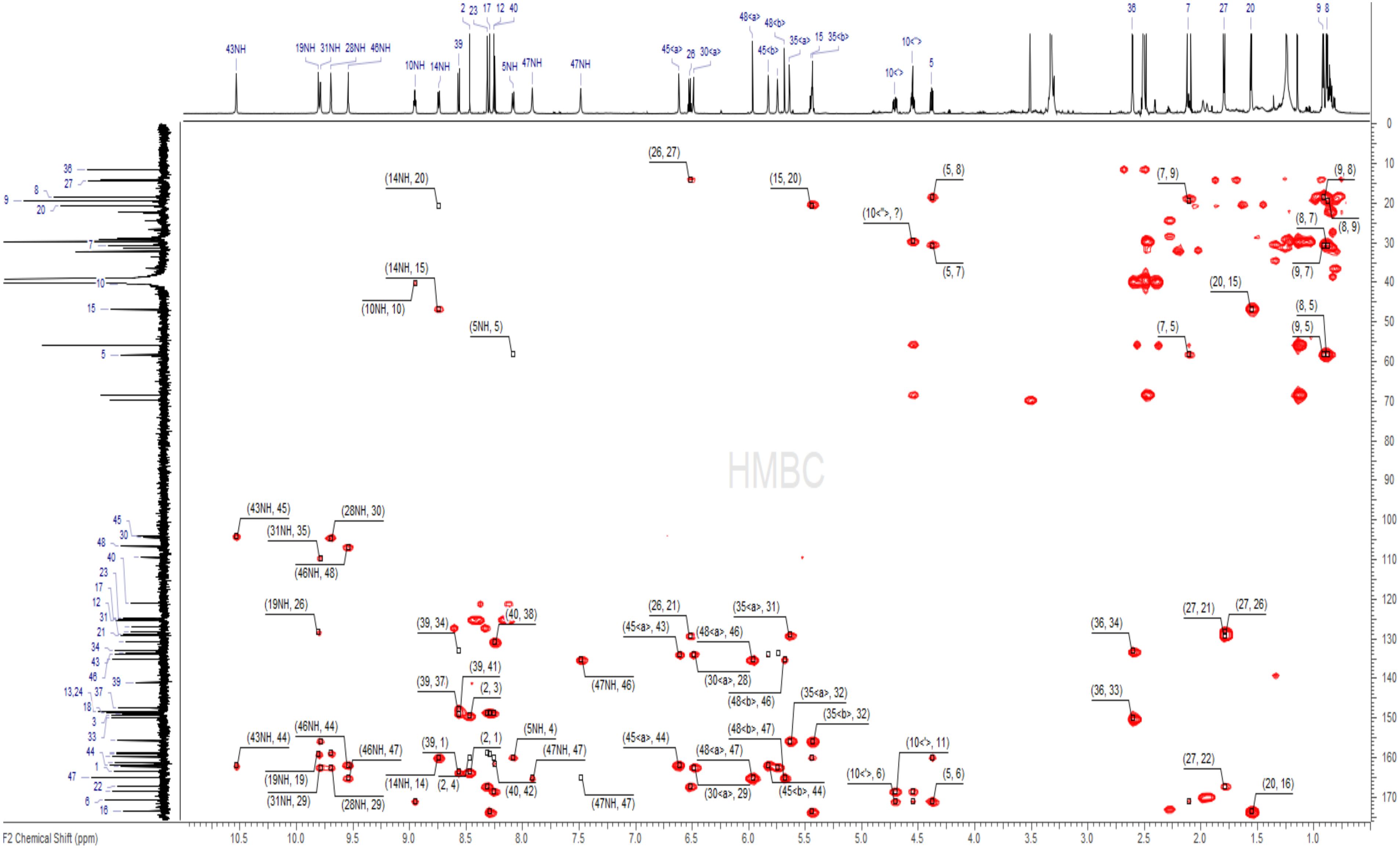

Figure S11: HMBC NMR spectrum (700 MHz, DMSO-d_6) of litoralimycin A (**1**).

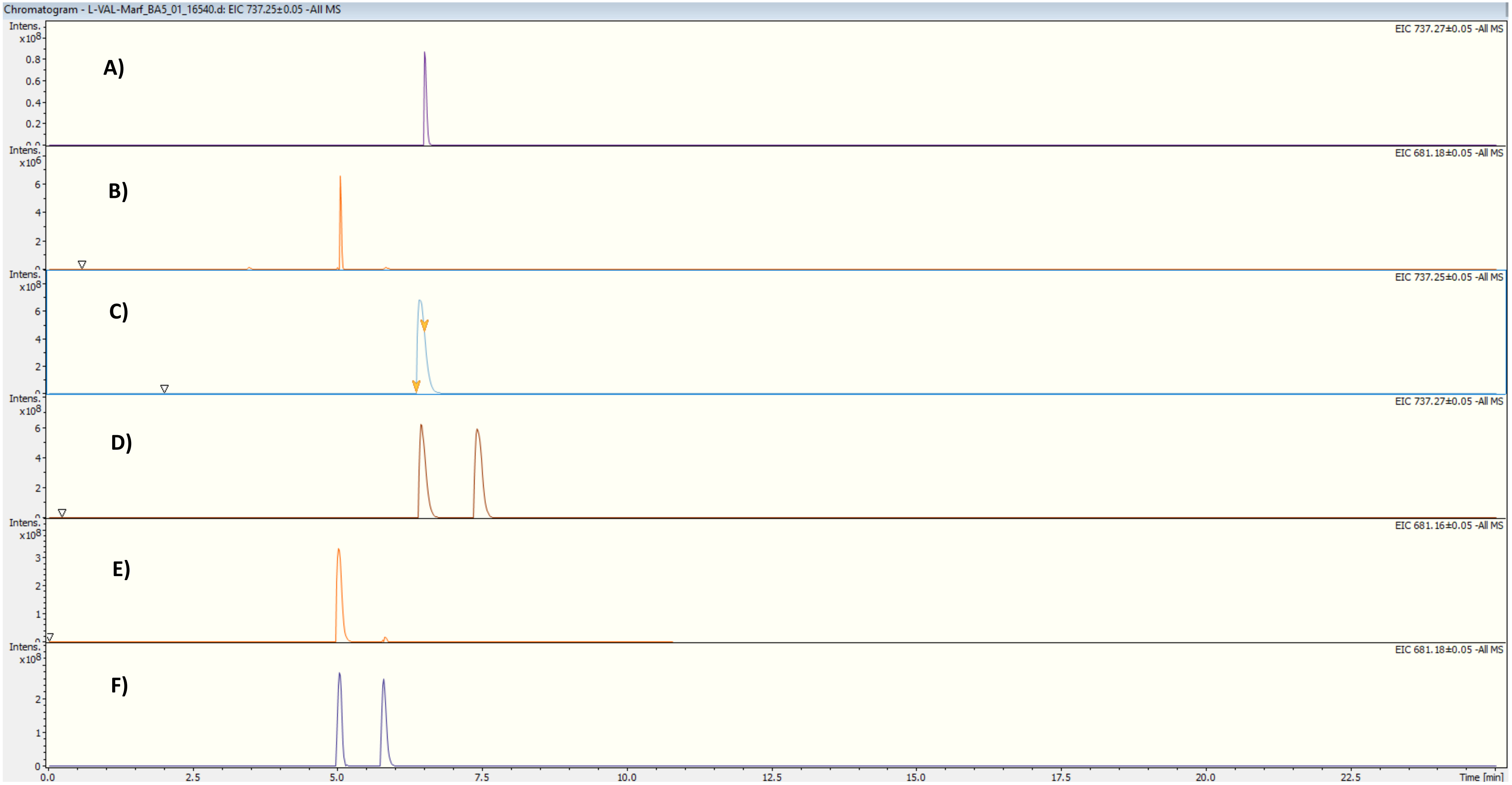

Figure S12: Marfey derivatization for determination of type and configuration of amino acids in litoralimycin A (**1**).

A) Detection of L-valine after ozonolysis, hydrolysis and derivatisation with FDAA; B) Detection of L-alanin after ozonolysis, hydrolysis and derivatisation with FDAA, C) FDAA derivative of L-Valin standard, D) FDAA derivative of DL-Valin standard, E) FDAA derivative of L-Alanin standard, F) FDAA derivative of DL-Alanin standard.

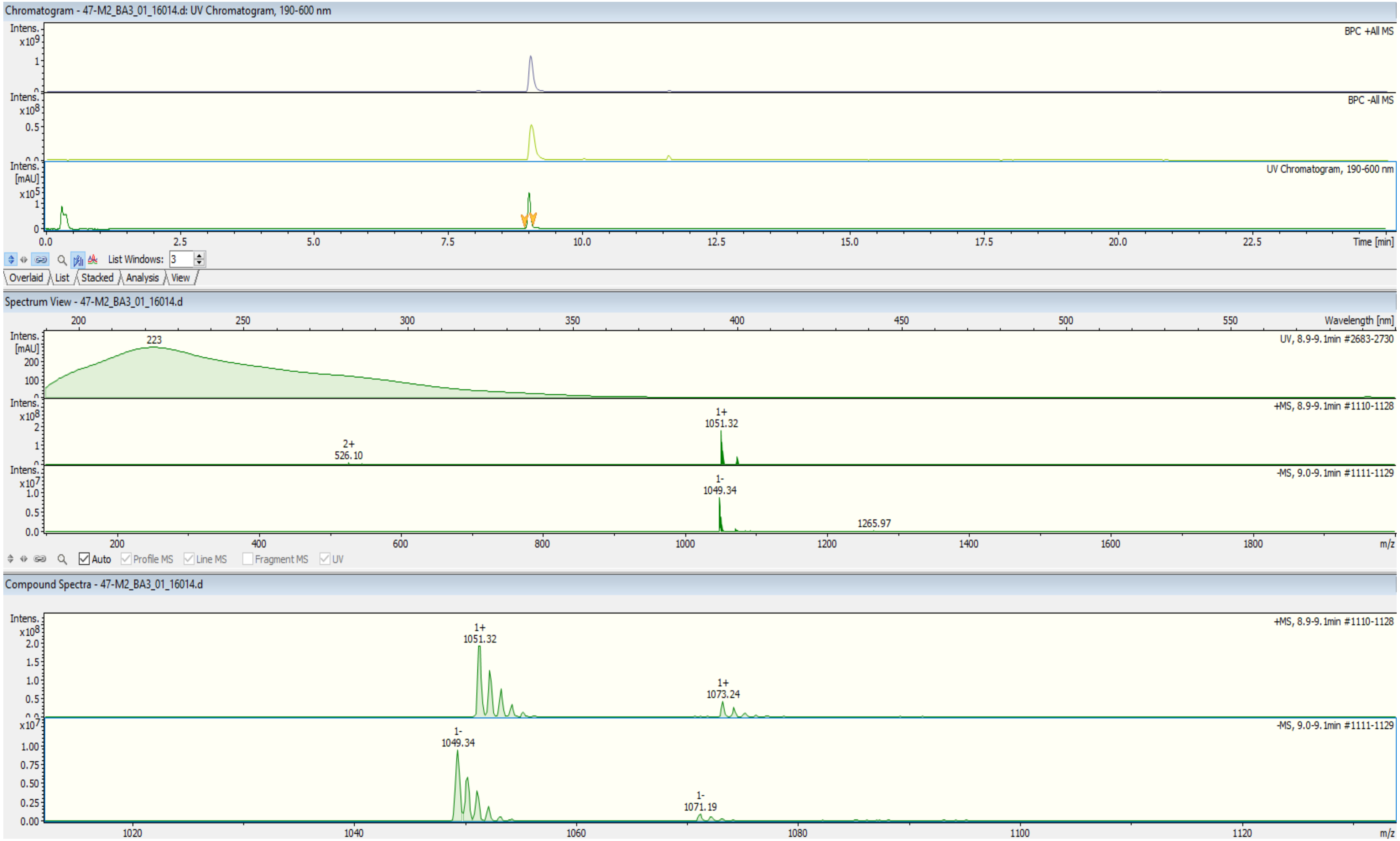

Figure S13: HPLC-ESIMS spectrum of litoralimycin B (**2**).

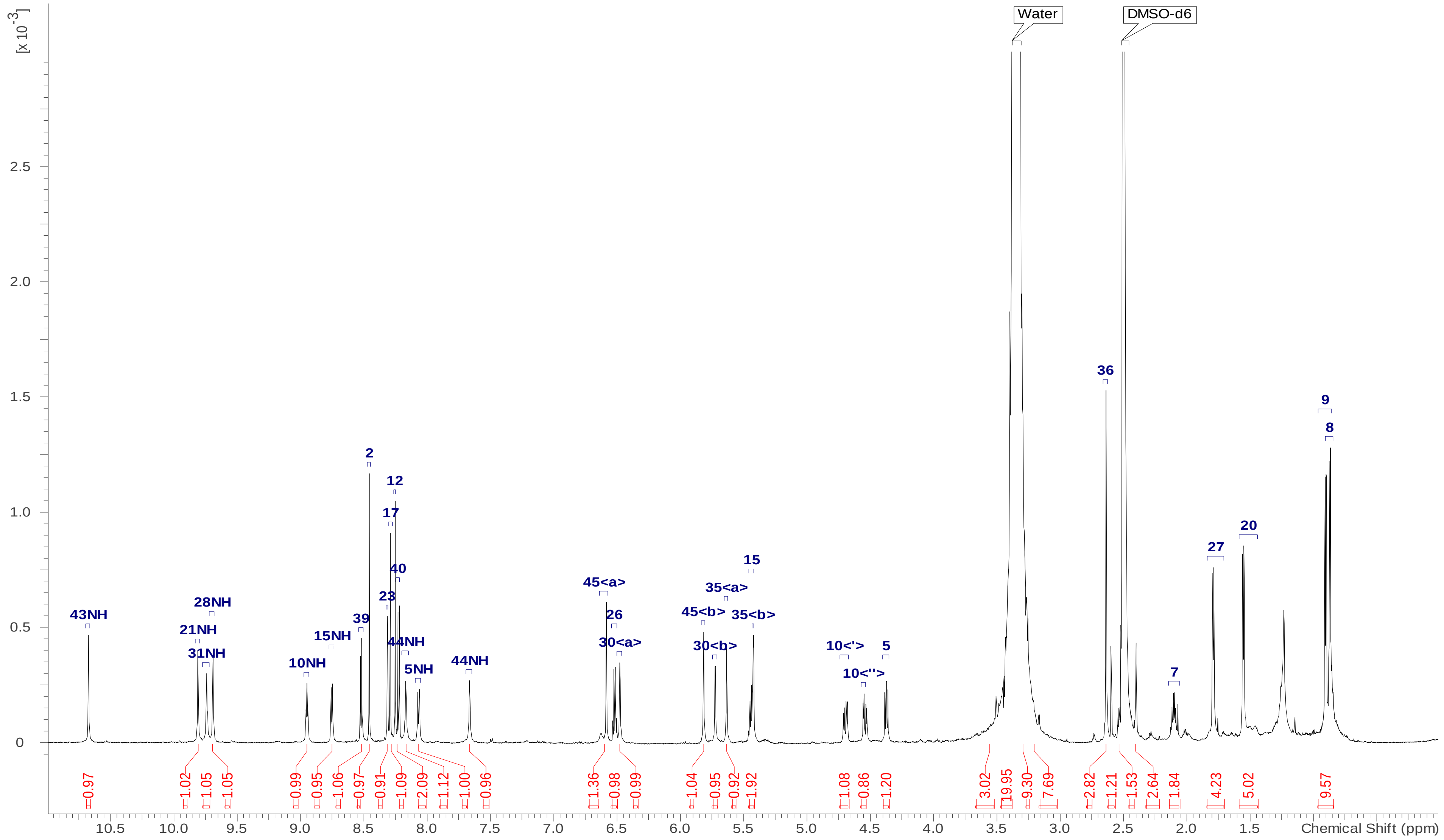

Figure S14: ^{1}H NMR spectrum (700 MHz, DMSO-d_6) of litoralimycin B (**2**).

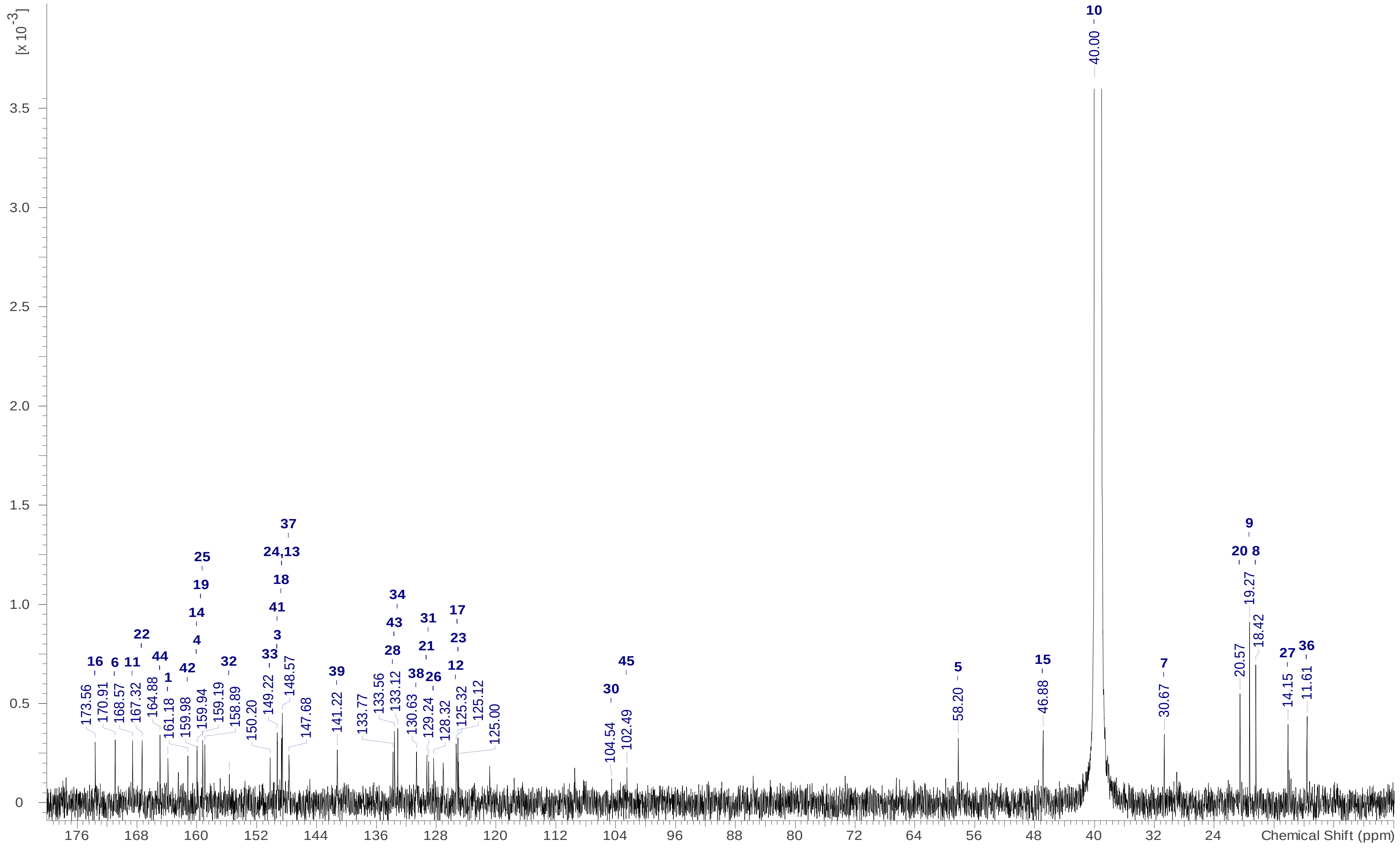

Figure S15: ^{13}C NMR spectrum (175 MHz, DMSO-d_6) of litoralimycin B (**2**).

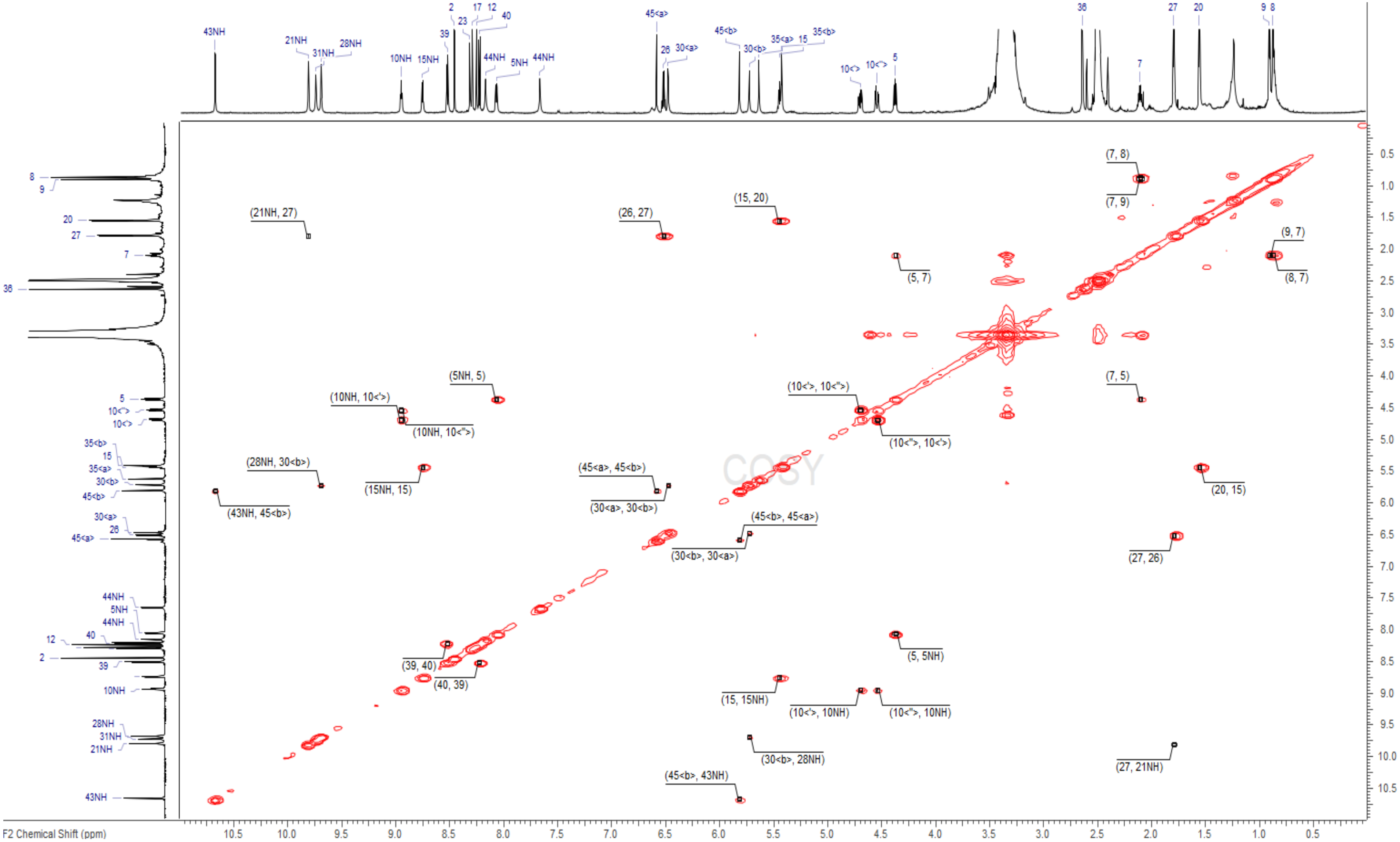

Figure S16: COSY NMR spectrum (700 MHz, DMSO-d_6) of litoralimycin B (**2**).

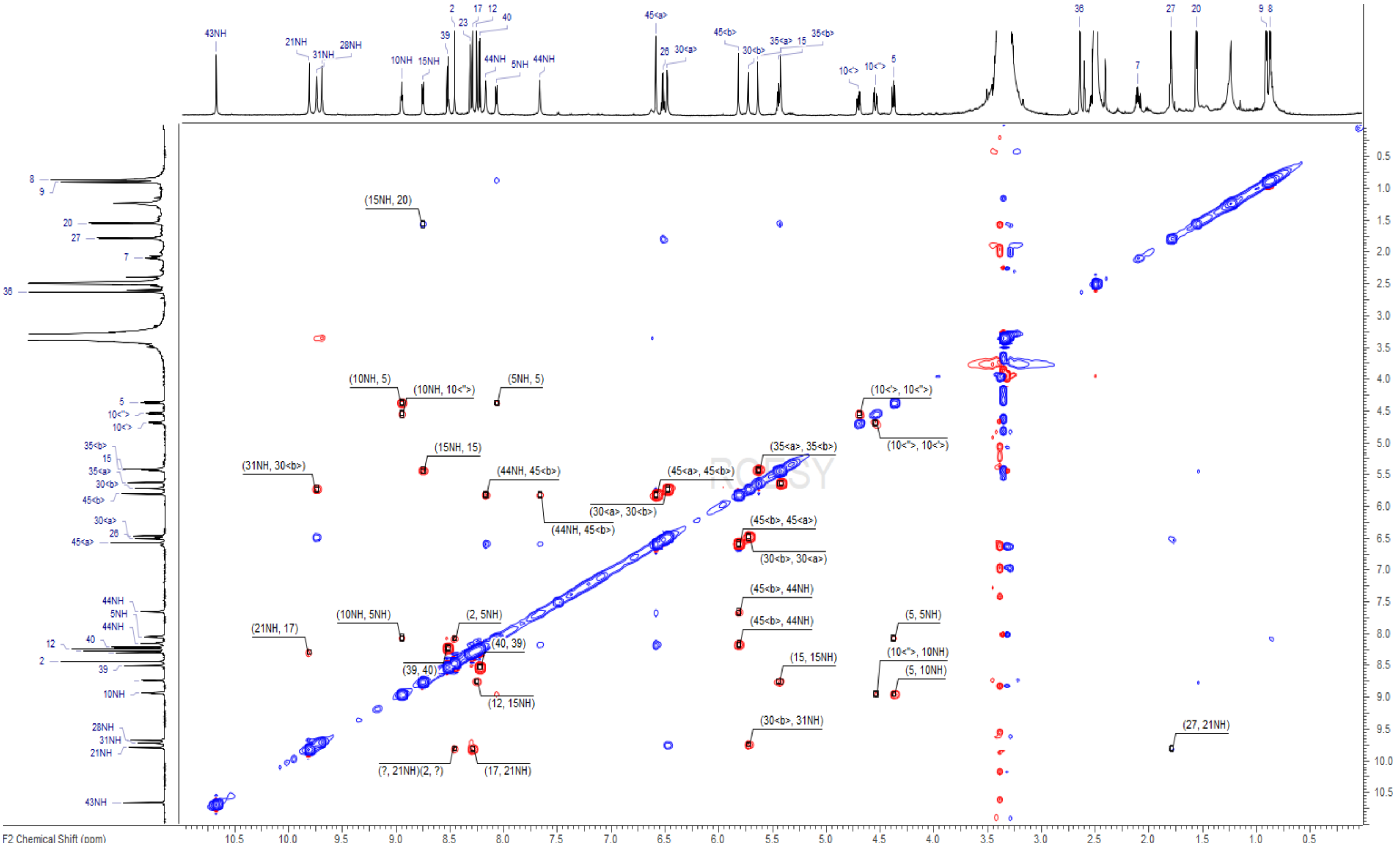

Figure S17: ROESY NMR spectrum (700 MHz, DMSO-d_6) of litoralimycin B (**2**).

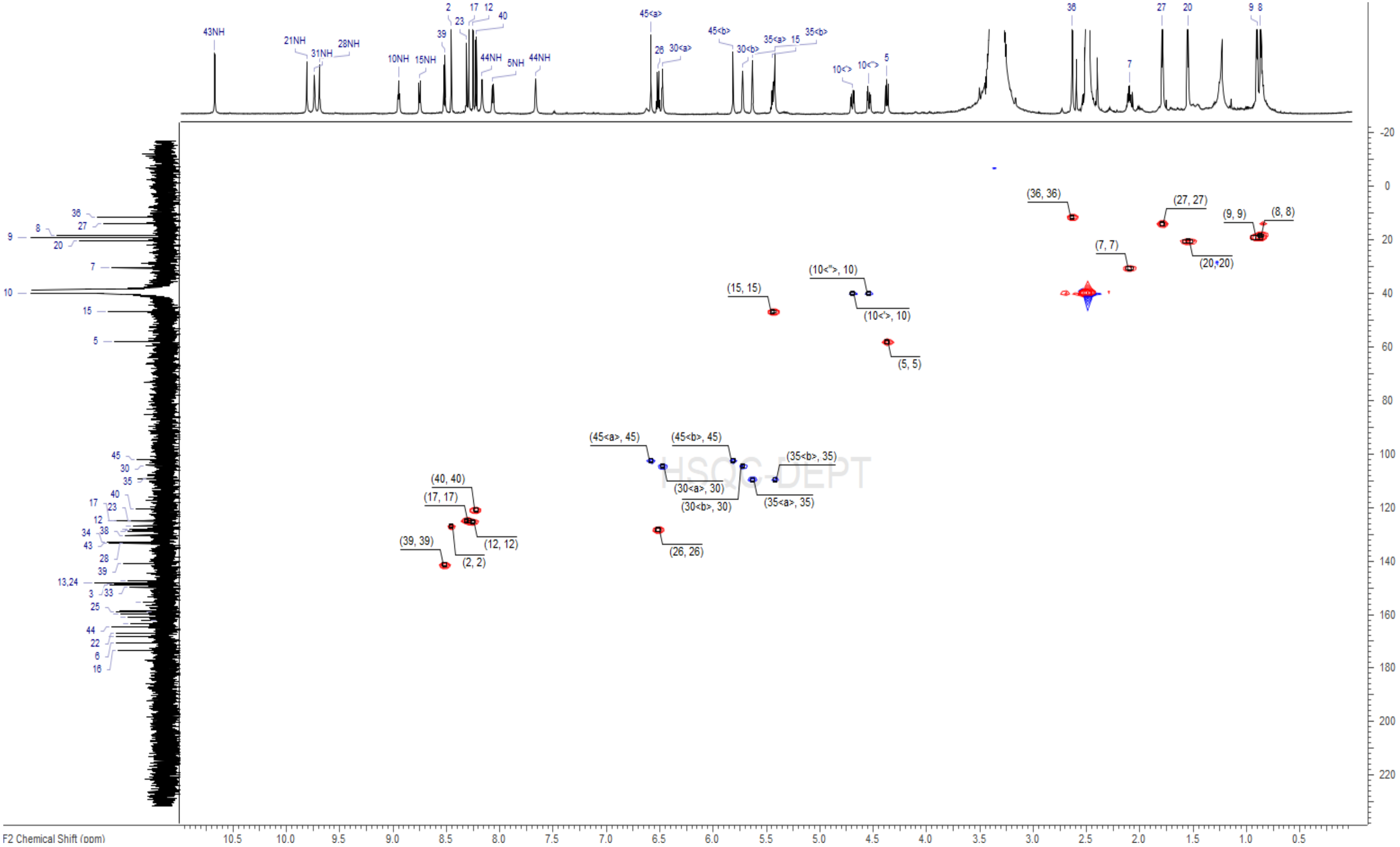

Figure S18: HSQC NMR spectrum (700 MHz, DMSO-d_6) of litoralimycin B (**2**).

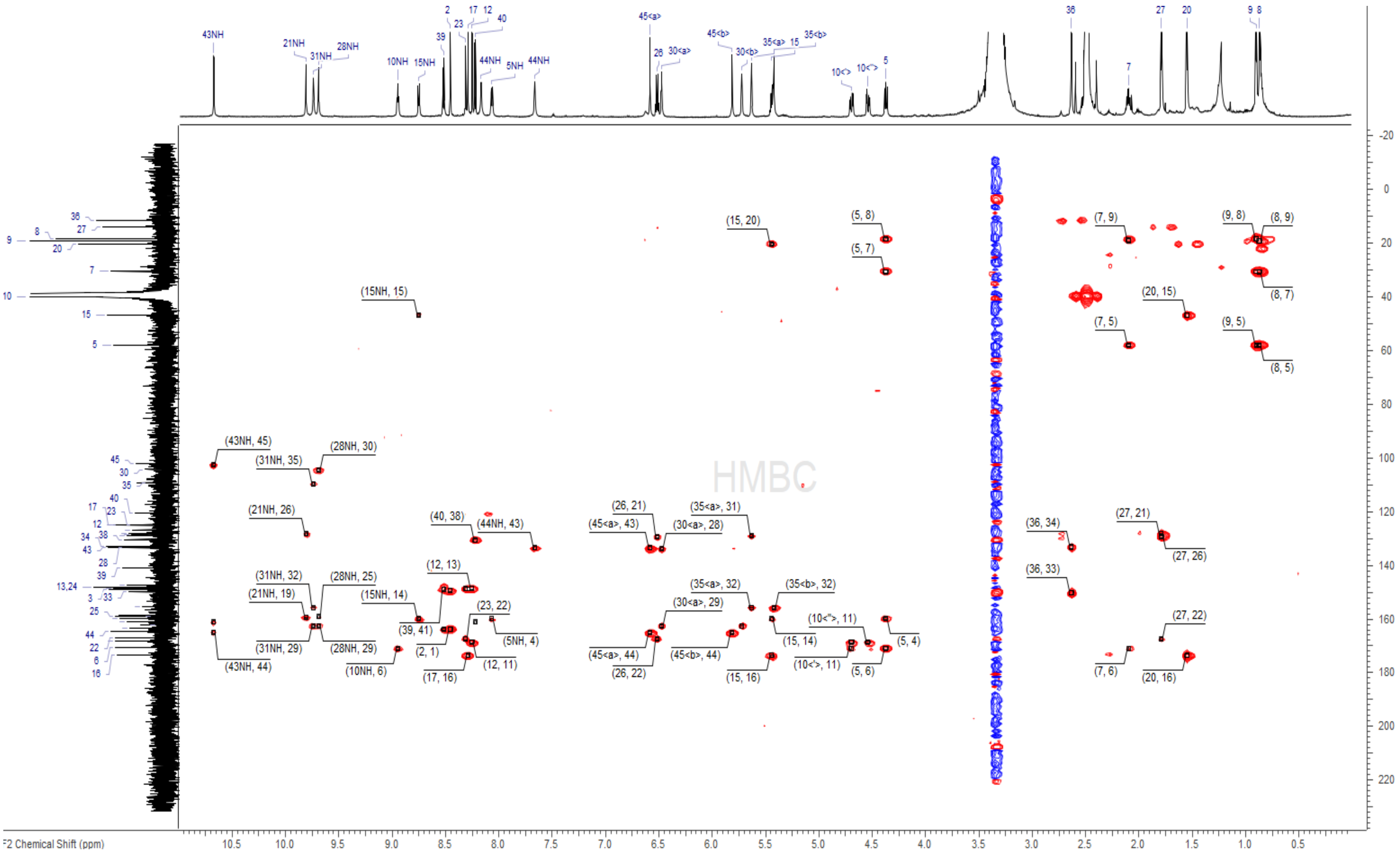

Figure S19: HMBC NMR spectrum (700 MHz, DMSO-d_6) of litoralimycin B (**2**).

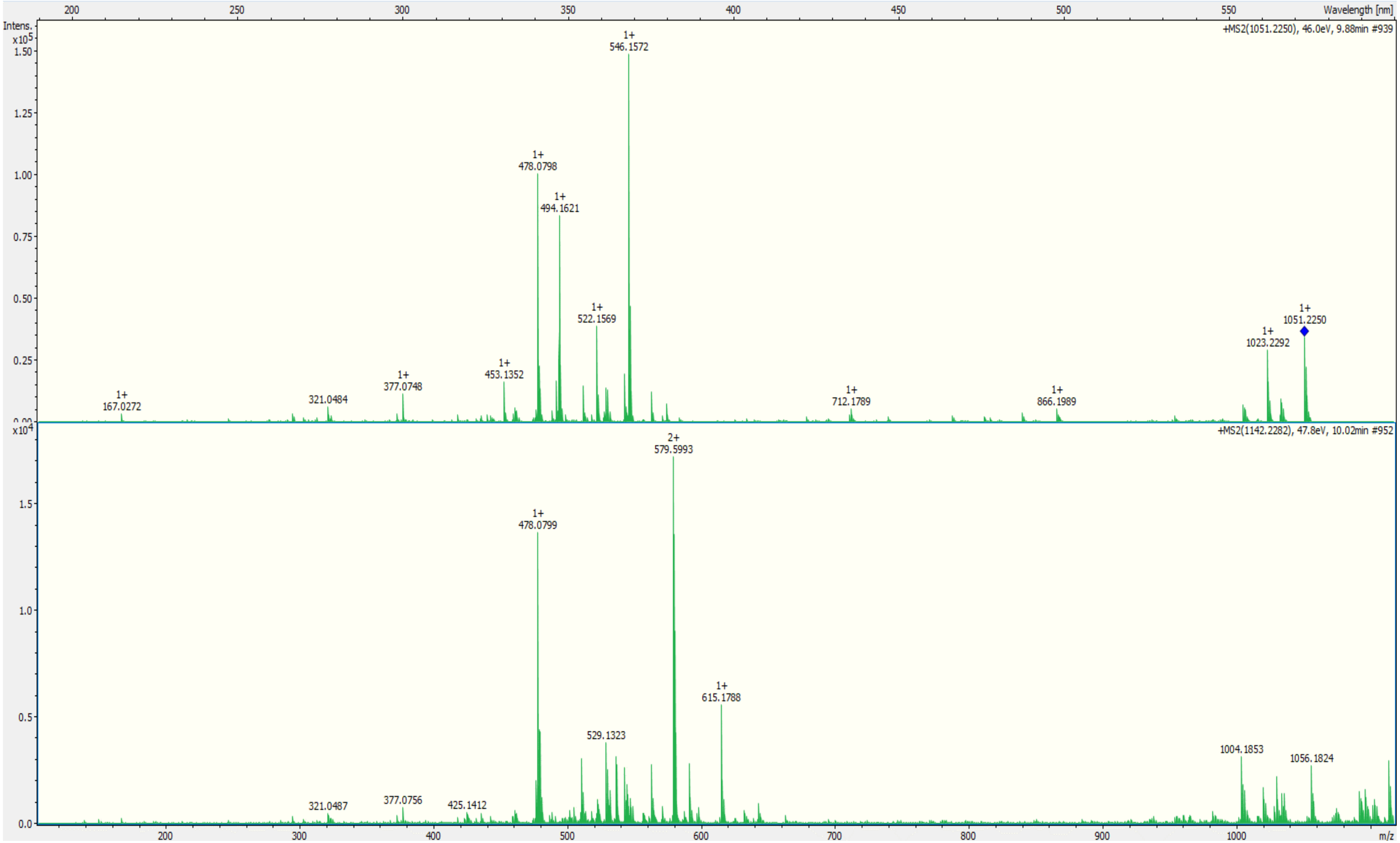

Figure 20: MS/MS data for **1** (lower part) and **2** (upper part) with fragmentation of $[M+Na]^+$ ions at *m/z* 1142.2282 and 1051.2250, respectively.
